# Supplementary material for: How much of the Mexican agricultural supply is produced by small farms, and how?
Source: PLoS One. 2023 Oct 5;18(10):e0292528. doi: 10.1371/journal.pone.0292528 (PMC10553241; doi:10.1371/journal.pone.0292528)
Supplement: S1 Table — (DOCX) [file pone.0292528.s001.docx]

**Supporting Information 1:**

Mexican agricultural production and domestic supply of the 29 agricultural products analysed in this study in million tonnes, for the year 2019, compared with the total values reported by the FAO. Source of data: calculations by the authors using FAO (2022c) for agricultural production values (data of “crops primary” and “livestock primary”), and FAO (2022a) for domestic supply values. Note that FAO does not give values for amaranth and alfalfa. Each food product of these table is associated with a food product analysed in this paper (see food product in italics after the semicolons). The details on the values with decimal points are shown in the Supporting Information 5.

**S1 Table. Agricultural production and domestic supply of the 29 agricultural products used in this study**

| **a. . Agricultural Production** **Food product (FAO, 2022c)** | **Production [Million tons**] |
| --- | --- |
| Maize (corn); *white & yellow maize* | 27.23 |
| Wheat; *wheat* | 3.24 |
| Rice; *rice* | 0.25 |
| *Amaranth* | no data |
| Beans, dry; *bean* | 0.88 |
| Pumpkins, squash and gourds; *squash* | 0.82 |
| Onions and shallots, dry (excluding dehydrated); *onion* | 1.49 |
| Chillies and peppers, green (Capsicum spp. and Pimenta spp.); *chilli pepper* | 2.72 |
| Tomatoes; *tomato* | 4.27 |
| Avocados; *avocado* | 2.30 |
| Strawberries; *strawberry* | 0.86 |
| Lemons and limes; *lemon* | 2.70 |
| Mangoes, guavas and mangosteens; *mango* | 2.40 |
| Apples; *apple* | 0.76 |
| Oranges; *orange* | 4.74 |
| Bananas; *banana* | 2.40 |
| Grapes; *grape* | 0.47 |
| Sorghum; *sorghum* | 4.35 |
| Soya beans; *soybean* | 0.23 |
| Raw milk of cattle; *milk* | 12.28 |
| Meat of cattle with the bone, fresh or chilled; *cattle meat* | 2.03 |
| Meat of pig with the bone, fresh or chilled; *pork* | 1.60 |
| Meat of chickens, fresh or chilled; *chicken meat* | 3.48 |
| Hen eggs in shell, fresh; *eggs* | 2.95 |
| Sugar cane; *sugar cane* | 59.33 |
| Coffee, green; *coffee* | 0.17 |
| Cocoa beans; *cocoa* | 0.03 |
| TOTAL for the 29 agricultural products included in this study: | 143.97 |
| Total "crops primary" and "livestock primary" reported by the FAO (2022c) | 166.97 |
| Share of the 29 food products included in this study in Mexico’s total agricultural production | 87% |

| **b. Domestic supply: Food product**  **(Food Balance Sheets: FAO, 2022a)** | **Domestic supply** [Million tons] |
| --- | --- |
| Wheat and products*; wheat* | 6.84 |
| Rice and products*; rice* | 1.41 |
| Maize and products; *white & yellow maize* | 47.13 |
| Beans*; bean* | 1.11 |
| Tomatoes and products*; tomato* | 2.56 |
| Onions*; onion* | 1.27 |
| Vegetables, other; *squash and chilli pepper* | 5.91 |
| Lemons, Limes and products*; lemon* | 1.73 |
| Apples and products*; apple* | 0.93 |
| Oranges, Mandarines; *orange* | 4.63 |
| Bananas; *banana* | 1.83 |
| Grapes and products (excl wine); *grape* | 0.39 |
| Fruits, other*; avocado, strawberry, mango,* | 6.11 |
| Sugar cane*; sugar cane* | 59.33 |
| Coffee and products*; coffee* | 0.08 |
| Cocoa Beans and products; *cocoa* | 0.09 |
| Sorghum and products*; sorghum* | 5.20 |
| Soyabeans*; soybeans* | 4.78 |
| Bovine Meat*; cattle meat* | 1.90 |
| Pigmeat*; pork* | 2.34 |
| Poultry Meat; *chicken meat* | 4.65 |
| Eggs*; eggs* | 3.00 |
| Milk – Excluding Butter; *eggs* | 13.49 |
| TOTAL amount in tonnes included in this study | 176.73 |
| Total domestic supply reported by the FAO (2022a) | 210.44 |
| Share of total included in this study | 84% |
