# Supplementary material for: How much of the Mexican agricultural supply is produced by small farms, and how?
Source: PLoS One. 2023 Oct 5;18(10):e0292528. doi: 10.1371/journal.pone.0292528 (PMC10553241; doi:10.1371/journal.pone.0292528)
Supplement: S2 Table — (DOCX) [file pone.0292528.s002.docx]

**Supporting information 2**

**Market and non-market production by each type of farm and each type of crop.** Calculations by the authors using INEGI (2019a)

The following tables show the share (per farm and crop) of the production and the total production in tons that was sold (“market”) and not sold (“no market”) by the farms. The “no market” production includes the seeds that farms use for the following farming season, the food used for home consumption, and the feed used for livestock. The details of the share and production for seeds, food and feed, as well as the values with decimal points, are shown in the Supporting Information.

**S2 Table.**

**a.** Total production of no market and market production produced by each type of farm and each type of crop. Source of data: calculations by the authors using INEGI (2019a)

| Tons | Small farms | | Medium farms | | Large farms | |
| --- | --- | --- | --- | --- | --- | --- |
|  | Not market | market | Not market | market | Not market | market |
| Avocado | 135 | 743,790 | 569 | 823,376 | 1,687 | 445,549 |
| Alfalfa | 863,822 | 1,694,021 | 171,608 | 1,324,157 | 484,387 | 1,070,391 |
| Amaranth | 8 | 567 | 69 | 1,020 | 223 | 3,466 |
| Rice | 868 | 107,858 | 183 | 39,057 | 23 | 9,443 |
| Cocoa | 46 | 3,229 | 1,192 | 5,407 | 1,749 | 12,644 |
| Coffee | 576 | 102,597 | 4,052 | 147,556 | 12,465 | 213,991 |
| Squash | 1,108 | 393,283 | 399 | 124,937 | 287 | 109,682 |
| Sugar Cane | 45,596 | 14,855,458 | 42,287 | 24,000,000 | 65,156 | 14,337,786 |
| Onion | 50 | 826,122 | 9 | 129,075 | 114 | 43,953 |
| Chilli pepper | 449 | 800,517 | 1,019 | 604,318 | 574 | 47,899 |
| Strawberry | - | 21,574 | 4 | 14,085 | 14 | 8,085 |
| Bean | 44,175 | 465,147 | 40,011 | 226,473 | 17,751 | 34,507 |
| Tomato | 907 | 893,758 | 1,265 | 121,576 | 162 | 57,949 |
| Lemon | 279 | 912,147 | 131 | 307,623 | 573 | 130,303 |
| Yellow maize | 521,165 | 3,500,598 | 247,125 | 694,573 | 197,026 | 240,494 |
| White maize | 1,231,565 | 10,724,702 | 1,152,651 | 8,453,322 | 1,261,406 | 2,987,188 |
| Mango | 325 | 473,256 | 2,584 | 253,098 | 1,152 | 184,598 |
| Apple | 157 | 222,431 | 5 | 147,663 | 223 | 22,376 |
| Orange | 1,370 | 1,617,578 | 3,261 | 1,232,379 | 3,148 | 642,371 |
| Banana | 438 | 1,499,405 | 555 | 140,201 | 1,296 | 285,591 |
| Sorghum | 193,156 | 2,199,371 | 56,589 | 869,511 | 13,056 | 339,386 |
| Soybean | 674 | 271,210 | 303 | 33,767 | 0 | 4,458 |
| Wheat | 16,881 | 3,328,673 | 2,338 | 367,088 | 280 | 19,831 |
| Grape | 47 | 283,532 | 15 | 20,745 | - | - |

**b.** Share of no market and market production produced by each type of farm and each type of crop

|  | Small farms | | Medium farms | | Large farms | |
| --- | --- | --- | --- | --- | --- | --- |
|  | No market | market | No market | market | No market | market |
| Avocado | 0.02% | 99.98% | 0.07% | 99.93% | 0.38% | 99.62% |
| Alfalfa | 33.77% | 66.23% | 11.47% | 88.53% | 31.15% | 68.85% |
| Amaranth | 1.33% | 98.67% | 6.33% | 93.67% | 6.04% | 93.96% |
| Rice | 0.80% | 99.20% | 0.47% | 99.53% | 0.24% | 99.76% |
| Cocoa | 1.39% | 98.61% | 18.06% | 81.94% | 12.15% | 87.85% |
| Coffee | 0.56% | 99.44% | 2.67% | 97.33% | 5.50% | 94.50% |
| Squash | 0.28% | 99.72% | 0.32% | 99.68% | 0.26% | 99.74% |
| Sugar Cane | 0.31% | 99.69% | 0.18% | 99.82% | 0.45% | 99.55% |
| Onion | 0.01% | 99.99% | 0.01% | 99.99% | 0.26% | 99.74% |
| Chilli pepper | 0.06% | 99.94% | 0.17% | 99.83% | 1.18% | 98.82% |
| Strawberry | 0.00% | 100.00% | 0.03% | 99.97% | 0.17% | 99.83% |
| Bean | 8.67% | 91.33% | 15.01% | 84.99% | 33.97% | 66.03% |
| Tomato | 0.10% | 99.90% | 1.03% | 98.97% | 0.28% | 99.72% |
| Lemon | 0.03% | 99.97% | 0.04% | 99.96% | 0.44% | 99.56% |
| Yellow maize | 12.96% | 87.04% | 26.24% | 73.76% | 45.03% | 54.97% |
| White maize | 10.30% | 89.70% | 12.00% | 88.00% | 29.69% | 70.31% |
| Mango | 0.07% | 99.93% | 1.01% | 98.99% | 0.62% | 99.38% |
| Apple | 0.07% | 99.93% | 0.00% | 100.00% | 0.99% | 99.01% |
| Orange | 0.08% | 99.92% | 0.26% | 99.74% | 0.49% | 99.51% |
| Banana | 0.03% | 99.97% | 0.39% | 99.61% | 0.45% | 99.55% |
| Sorghum | 8.07% | 91.93% | 6.11% | 93.89% | 3.70% | 96.30% |
| Soybean | 0.25% | 99.75% | 0.89% | 99.11% | 0.00% | 100.00% |
| Wheat | 0.50% | 99.50% | 0.63% | 99.37% | 1.39% | 98.61% |
| Grape | 0.02% | 99.98% | 0.07% | 99.93% | 0.00% | 0.00% |
