# Supplementary material for: How much of the Mexican agricultural supply is produced by small farms, and how?
Source: PLoS One. 2023 Oct 5;18(10):e0292528. doi: 10.1371/journal.pone.0292528 (PMC10553241; doi:10.1371/journal.pone.0292528)
Supplement: S3 Table — (DOCX) [file pone.0292528.s003.docx]

**Supporting information 3**

**Imports and exports of agricultural products estimated for this study**. Calculations by the authors using INEGI (2019a) and FAO (2022b).

The FAO gives data of exports and imports (FAO, 2022b) and production values (FAO, 2022c) of all primary products. The values of production (FAO, 2022c) are slightly different from the values reported by the INEGI (2019a) (values of this paper) due to differences in methodology and type of agricultural products used in each data source. For this reason, for this study we calculated a “ratio value” of exports and imports using FAO data (Supplementary Information 2). This ratio was used to calculate the exports and imports for the 29 food groups of this study The following table shows the results of this calculation.

The FAO does not distinguish between white and yellow maize. So, yellow and white maize production and trade values were taken from SAGARPA (2017). The details on the values with decimal points are shown in the Supporting Information.

**S3 Table**

a. **Estimation of exports and imports ratio value.** Each food product of these table is associated with a food product analysed in this paper (see food product in italics after the semicolons). Data source: Production (FAO, 2022c), imports and exports (FAO 2022b).

| **Food product**  **(FAO: 2022b,2022c)** | Production  (FAO, 2022c) | Exports  (FAO 2022b) | Imports  (FAO 2022b) | Ratio of exports | Ratio of imports |
| --- | --- | --- | --- | --- | --- |
| *White maize* | 24.56 | 1.499 | 1.066 | 0.06 | 0.04 |
| Wheat*; wheat* | 3.24 | 0.74 | 4.80 | 0.23 | 1.48 |
| Rice, paddy (rice milled eq)*; rice* | 0.16 | 0.01 | 0.97 | 0.06 | 5.95 |
| *Amaranth* | no data | no data | no data | - | - |
| beans, dry*; bean* | 0.88 | 0.04 | 0.12 | 0.05 | 0.14 |
| sugar cane*; sugar cane* | no data | no data | no data | - | - |
| Pumpkins, squash and gourds*; squash* | 0.68 | 0.54 | 0.00 | 0.79 | - |
| Onions, dry*; onion* | 1.49 | 0.35 | 0.13 | 0.24 | 0.09 |
| Chillies and peppers, green*; chili pepper* | 3.24 | 1.07 | 0.00 | 0.33 | 0.00 |
| Tomatoes; *tomato* | 4.27 | 1.86 | 0.00 | 0.44 | 0.00 |
| Avocados*; avocado* | 2.30 | 1.15 | 0.00 | 0.50 | - |
| Strawberries; *strawberry* | 0.86 | 0.14 | 0.02 | 0.16 | 0.02 |
| Lemons and limes*; lemon* | 2.70 | 0.77 | 0.00 | 0.28 | 0.00 |
| Mangoes, mangosteens, guavas*; mango* | 2.40 | 0.41 | 0.00 | 0.17 | 0.00 |
| Apples*; apple* | 0.76 | 0.00 | 0.25 | 0.00 | 0.33 |
| Oranges*; orange* | 4.74 | 0.06 | 0.03 | 0.01 | 0.01 |
| Bananas*; banana* | 2.40 | 0.57 | 0.00 | 0.24 | 0.00 |
| Grapes*; grape* | 0.47 | 0.22 | 0.09 | 0.46 | 0.19 |
| Cocoa, beans*; cocoa* | 0.03 | 0.00 | 0.05 | 0.00 | 1.64 |
| Coffee, green*; coffee* | 0.17 | 0.10 | 0.04 | 0.59 | 0.24 |
| *Yellow maize* | 3.55 | 0.035 | 12.95 | 0.01 | 3.65 |
| Alfalfa meal and pellets*; alfalfa* | 0.0 | 0.0 | 0.0 | 0.0 | 0.0 |
| Sorghum*; sorghum* | 4.35 | 0.00 | 0.74 | 0.00 | 0.17 |
| Soybeans*; soybean* | 0.23 | 0.00 | 4.85 | 0.01 | 20.85 |
| Milk, whole fresh cow*; milk* | 12.28 | 0.00 | 0.21 | 0.00 | 0.02 |
| Meat, cattle; *cattle meat* | 2.03 | 0.10 | 0.00 | 0.05 | 0.00 |
| Meat, pig*; pork* | 1.60 | 0.00 | 0.70 | 0.00 | 0.44 |
| Meat, chicken*; chicken meat* | 3.48 | 0.00 | 0.97 | - | 0.28 |
| Eggs, hen, in shell*; eggs* | 2.95 | 0.00 | 0.05 | - | 0.02 |

**b.** **Production, exports, imports, and domestic supply values.** Data source: Calculations by the authors.

| **[Million tons]** | Production | Exports | Imports | Domestic Supply |
| --- | --- | --- | --- | --- |
| White maize | 25.81 | 1.58 | 1.12 | 25.36 |
| Wheat | 3.74 | 0.85 | 5.53 | 8.42 |
| Rice | 0.16 | 0.01 | 0.94 | 1.08 |
| Amaranth | 0.01 | 0.00 | 0.00 | 0.01 |
| Bean | 0.83 | 0.04 | 0.12 | 0.90 |
| Sugar cane | 53.25 | 0.00 | 0.00 | 53.25 |
| Squash | 0.64 | 0.50 | 0.00 | 0.13 |
| Onion | 1.00 | 0.24 | 0.09 | 0.85 |
| Chilli pepper | 2.00 | 0.66 | 0.00 | 1.34 |
| Tomato | 2.86 | 1.24 | 0.00 | 1.62 |
| Avocado | 2.02 | 1.01 | 0.00 | 1.01 |
| Strawberry | 0.23 | 0.04 | 0.00 | 0.20 |
| Lemon | 1.35 | 0.38 | 0.00 | 0.97 |
| Mango | 0.92 | 0.16 | 0.00 | 0.76 |
| Apple | 0.48 | 0.00 | 0.16 | 0.64 |
| Orange | 3.50 | 0.04 | 0.02 | 3.48 |
| Banana | 1.93 | 0.46 | 0.00 | 1.47 |
| Grape | 0.32 | 0.15 | 0.06 | 0.23 |
| Cocoa | 0.02 | 0.00 | 0.04 | 0.06 |
| Coffee | 0.48 | 0.29 | 0.12 | 0.31 |
| Yellow maize grain | 5.40 | 0.05 | 19.70 | 25.05 |
| Alfalfa | 5.61 | 0.00 | 0.00 | 5.61 |
| Sorghum | 3.67 | 0.00 | 0.63 | 4.30 |
| Soybean | 0.31 | 0.00 | 6.48 | 6.78 |
| Milk | 12.49 | 0.00 | 0.21 | 12.70 |
| Cattle meat | 2.03 | 0.10 | 0.00 | 1.93 |
| Pork | 1.60 | 0.00 | 0.70 | 2.30 |
| Chicken meat | 3.48 | 0.00 | 0.97 | 4.45 |
| Eggs | 2.95 | 0.00 | 0.05 | 3.00 |
| TOTAL | 139.08 | 7.80 | 36.95 | 168.22 |
