# Supplementary material for: How much of the Mexican agricultural supply is produced by small farms, and how?
Source: PLoS One. 2023 Oct 5;18(10):e0292528. doi: 10.1371/journal.pone.0292528 (PMC10553241; doi:10.1371/journal.pone.0292528)
Supplement: S4 Table — (DOCX) [file pone.0292528.s004.docx]

**Supporting information 4**.

**Distribution of agricultural products production by type of farm in Mexico.**

Values are given in million tons, the share of the production per type of farms indicated in parenthesis. Source: calculations by the authors. See section 3.1 for details.

**S4 Table**  **Distribution of agricultural products production by type of farm in Mexico.**

| Mt (%) | Small scale | Medium scale | Large scale | Protected agriculture | Total |
| --- | --- | --- | --- | --- | --- |
| White maize | 4.0  (17%) | 9.0  (37%) | 11.2  (46%) | 0.0  (0%) | 24.2  (100%) |
| Other cereals | 0.0  (1%) | 0.3  (11%) | 2.7  (88%) | 0.0  (0%) | 3.0  (100%) |
| Beans | 0.0  (6%) | 0.3  (32%) | 0.5  (62%) | 0.0  (0%) | 0.8  (100%) |
| Vegetables | 0.1  (3%) | 0.6  (15%) | 1.8  (46%) | 1.4  (36%) | 3.8  (100%) |
| Fruits | 1.4  (16%) | 2.3  (28%) | 4.6  (53%) | 0.2  (3%) | 8.5  (100%) |
| Animal feed | 2.3  (16%) | 3.4  (23%) | 9.2  (61%) | 0.0  (0%) | 14.9  (100%) |
| Animal products | 3.2  (14%) | 3.9  (18%) | 15.3  (68%) | N.A. | 22.4  (100%) |
| Stimulants | 0.1  (48%) | 0.1  (31%) | 0.0  (20%) | 0.0  (1%) | 0.2  (100%) |
| Sugar cane | 14.3  (27%) | 24.0  (45%) | 14.9  (28%) | 0.0  (0%) | 53.3  (100%) |
| TOTAL | 25.6  (19%) | 43.9  (33%) | 60.1  (46%) | 1.6  (2%) | 131.3  (100%) |
