# Supplementary material for: How much of the Mexican agricultural supply is produced by small farms, and how?
Source: PLoS One. 2023 Oct 5;18(10):e0292528. doi: 10.1371/journal.pone.0292528 (PMC10553241; doi:10.1371/journal.pone.0292528)
Supplement: Attachment — Submitted filename: TNPI-04-09-2023-PONE-D-23-16291_R1_reviewer.pdf [file pone.0292528.s008.pdf]

PLOS ONE

How much of the Mexican agricultural supply is produced by small farms, and how?

--Manuscript Draft--

**Manuscript Number:**	PONE-D-23-16291R1
**Article Type:**	Research Article
**Full Title:**	How much of the Mexican agricultural supply is produced by small farms, and how?
**Short Title:**	How much of the Mexican agricultural supply is produced by small farms, and how?
**Corresponding Author:**	Maria-Jose Ibarrola Ibarrola-Rivas, PhD<br>Universidad Nacional Autonoma de Mexico Instituto de Geografia<br>Ciudad de México, Del. Coyoacán MEXICO
**Keywords:**	Agricultural supply; small farms; Mexico; farming practices; peasants
**Abstract:**	The contribution of small farms to the global food supply is in debate due to lack of empirical evidence. In Mexico, small farms have been relatively important for national food supply due to an agrarian reform in the first half of the 20th century, but their role has been decreasing in the last decades. The aim of this study is to quantify how much small farms produce of the Mexican agricultural supply, and with which farming practices, using the 2019 National Agricultural Survey. The results show that small farms produce 19% of the national agricultural production with similar farming practices to those of medium and large farms. When considering imports and exports, small farms produce 15% of the national agricultural supply. The production of small farms consists mainly of cash crops (e.g. sugar cane, fruits & vegetables, animal products, fodder crops) and, to a lesser extent, staple crops such as maize and beans. The fact that small farms produce one fifth of the national production after decades of governmental support towards large farms suggests that they have resilient production systems. The results of this study support that stronger efforts should be made to enhance the role of small farms in achieving Mexican food sovereignty. This will not only have benefits in terms of food supply but may also have a wide range of social and environmental benefits.
**Order of Authors:**	Maria-Jose Ibarrola Ibarrola-Rivas, PhD
	Quetzalcóatl Orozco-Ramírez, Dr
	Louise Guibrunet, PhD
**Response to Reviewers:**	See attached file
**Additional Information:**	
**Question**	**Response**
**Financial Disclosure**<br><br>Enter a financial disclosure statement that describes the sources of funding for the work included in this submission. Review the submission guidelines for detailed requirements. View published research articles from *PLOS ONE* for specific examples.<br><br>This statement is required for submission and **will appear in the published article** if the submission is accepted. Please make sure it is accurate.	This project was partly funded by two PAPIIT (Programa de Apoyo a Proyectos de Investigación e Innovación Tecnológica) projects of the National Autonomous University of Mexico with projects numbers: IN300322 for Ibarrola-Rivas and IA300222 for Guibrunet. https://dgapa.unam.mx/index.php/impulso-a-la-investigacion/papiit The funders had no role in study design, data collection and analysis, decision to publish, or preparation of the manuscript.

Powered by Editorial Manager® and ProduXion Manager® from Aries Systems Corporation

**Unfunded studies**<br>Enter: *The author(s) received no specific funding for this work.*<br><br>**Funded studies**<br>Enter a statement with the following details:<br>• Initials of the authors who received each award<br>• Grant numbers awarded to each author<br>• The full name of each funder<br>• URL of each funder website<br>• Did the sponsors or funders play any role in the study design, data collection and analysis, decision to publish, or preparation of the manuscript?<br>• **NO** - Include this sentence at the end of your statement: *The funders had no role in study design, data collection and analysis, decision to publish, or preparation of the manuscript.*<br>• **YES** - Specify the role(s) played.<br><br>* typeset	
**Competing Interests**<br><br>Use the instructions below to enter a competing interest statement for this submission. On behalf of all authors, disclose any competing interests that could be perceived to bias this work—acknowledging all financial support and any other relevant financial or non-financial competing interests.<br><br>This statement is required for submission and **will appear in the published article** if the submission is accepted. Please make sure it is accurate and that any funding sources listed in your Funding Information later in the submission form are also declared in your Financial Disclosure statement.<br><br>View published research articles from *PLOS ONE* for specific examples.	The authors have declared that no competing interests exist.

**NO authors have competing interests**<br><br>Enter: *The authors have declared that no competing interests exist.*<br><br>**Authors with competing interests**<br><br>Enter competing interest details beginning with this statement:<br><br>*I have read the journal's policy and the authors of this manuscript have the following competing interests: [insert competing interests here]*<br><br>* typeset	
**Ethics Statement**<br><br>Enter an ethics statement for this submission. This statement is required if the study involved:<br><br>• Human participants<br>• Human specimens or tissue<br>• Vertebrate animals or cephalopods<br>• Vertebrate embryos or tissues<br>• Field research<br><br>Write "N/A" if the submission does not require an ethics statement.<br><br>General guidance is provided below. Consult the submission guidelines for detailed instructions. **Make sure that all information entered here is included in the Methods section of the manuscript.**	N/A

**Format for specific study types**<br><br>**Human Subject Research (involving human participants and/or tissue)**<br>• Give the name of the institutional review board or ethics committee that approved the study<br>• Include the approval number and/or a statement indicating approval of this research<br>• Indicate the form of consent obtained (written/oral) or the reason that consent was not obtained (e.g. the data were analyzed anonymously)<br><br>**Animal Research (involving vertebrate animals, embryos or tissues)**<br>• Provide the name of the Institutional Animal Care and Use Committee (IACUC) or other relevant ethics board that reviewed the study protocol, and indicate whether they approved this research or granted a formal waiver of ethical approval<br>• Include an approval number if one was obtained<br>• If the study involved *non-human primates*, add *additional details* about animal welfare and steps taken to ameliorate suffering<br>• If anesthesia, euthanasia, or any kind of animal sacrifice is part of the study, include briefly which substances and/or methods were applied<br><br>**Field Research**<br><br>Include the following details if this study involves the collection of plant, animal, or other materials from a natural setting:<br>• Field permit number<br>• Name of the institution or relevant body that granted permission	
**Data Availability**<br><br>Authors are required to make all data underlying the findings described fully available, without restriction, and from the time of publication. PLOS allows rare exceptions to address legal and ethical concerns. See the PLOS Data Policy and FAQ for detailed information.	Yes - all data are fully available without restriction

A Data Availability Statement describing where the data can be found is required at submission. Your answers to this question constitute the Data Availability Statement and **will be published in the article**, if accepted.<br><br>**Important:** Stating 'data available on request from the author' is not sufficient. If your data are only available upon request, select 'No' for the first question and explain your exceptional situation in the text box.<br><br>Do the authors confirm that all data underlying the findings described in their manuscript are fully available without restriction?	
**Describe where the data may be found in full sentences. If you are copying our sample text, replace any instances of XXX with the appropriate details.**<br><br>• If the data are **held or will be held in a public repository**, include URLs, accession numbers or DOIs. If this information will only be available after acceptance, indicate this by ticking the box below. For example: *All XXX files are available from the XXX database (accession number(s) XXX, XXX.*).<br>• If the data are all contained **within the manuscript and/or Supporting Information files**, enter the following: *All relevant data are within the manuscript and its Supporting Information files.*<br>• If neither of these applies but you are able to provide **details of access elsewhere**, with or without limitations, please do so. For example:<br><br>*Data cannot be shared publicly because of [XXX]. Data are available from the XXX Institutional Data Access / Ethics Committee (contact via XXX) for researchers who meet the criteria for access to confidential data.*<br><br>*The data underlying the results presented in the study are available from (include the name of the third party*	All relevant data are within the manuscript and its Supporting Information files.

and contact information or URL).<br>• This text is appropriate if the data are owned by a third party and authors do not have permission to share the data.<br><br>* typeset	
Additional data availability information:	Tick here if the URLs/accession numbers/DOIs will be available only after acceptance of the manuscript for publication so that we can ensure their inclusion before publication.

Powered by Editorial Manager® and ProduXion Manager® from Aries Systems Corporation

Manuscript

Click here to access/download;Manuscript;Manuscrito_clean.docx

Title: How much of the Mexican agricultural supply is produced by small farms, and how?

Authors: María-José Ibarrola-Rivas [1, a]; Quetzalcóatl Orozco-Ramírez [2]; Louise Guibrunet [3]

Authors information:

[1] Physics Geography Department, Geography Institute, Autonomous National University of Mexico (UNAM), Mexico City, Mexico. ibarrola@geografia.unam.mx ORCID: 0000-0003-4501-0408

[2] Academic Unit campus Oaxaca, Geography Institute, Autonomous National University of Mexico (UNAM), Oaxaca, México. qorozco@geografia.unam.mx ORCID: 0000-0002-3085-7406

[3] Social Geography Department, Geography Institute, Autonomous National University of Mexico (UNAM), Mexico City, Mexico louiseg@geografia.unam.mx ORCID 0000-0002-1377-4852

Corresponding author: [a] Post Address: Institute of Geography UNAM. Circuito de la Investigación Científica S.N., Ciudad Universitaria, Coyoacán, C.P. 04510 Ciudad de México, CDMX

Acknowledgements The data used in this paper were processed and analyzed under project number LM-530 at the Microdata Laboratory of the National Institute of Statistics and Geography (INEGI) at their offices in Mexico City. The authors give special thanks to the staff of INEGI Microdata Laboratory (María de Lourdes Garrido Blancas, Lidia Hernández Hernández, Liliana Martínez Matias, Andrea López Ortiz, and Natalia Eugenia Volkow Fernández) and the experts of the agricultural census and survey at INEGI (Victor Dueñas de la Rosa, Karina Lara Fernández, Luis Fernando Esteves Cano, Ismael Saucedo Silva) for their unconditional support during our work at the Microdata Laboratory, even in times of pandemic.

Funding This project was partly funded by two PAPIIT (*Programa de Apoyo a Proyectos de Investigación e Innovación Tecnológica*) projects of the National Autonomous University of Mexico with projects numbers: IN300322 for Ibarrola-Rivas and IA300222 for Guibrunet. The funders had no role in study design, data collection and analysis, decision to publish, or preparation of the manuscript.

How much of the Mexican agricultural supply is produced by small farms, and how?

Abstract

The contribution of small farms to the global food supply is in debate due to lack of empirical evidence. In Mexico, small farms have been relatively important for national food supply due to an agrarian reform in the first half of the 20th century, but their role has been decreasing in the last decades. The aim of this study is to quantify how much small farms produce of the Mexican agricultural supply, and with which farming practices, using the 2019 National Agricultural Survey. The results show that small farms produce 19% of the national agricultural production with similar farming practices to those of medium and large farms. When considering imports and exports, small farms produce 15% of the national agricultural supply. The production of small farms consists mainly of cash crops (e.g. sugar cane, fruits & vegetables, animal products, fodder crops) and, to a lesser extent, staple crops such as maize and beans. The fact that small farms produce one fifth of the national production after decades of governmental support towards large farms suggests that they have resilient production systems. The results of this study support that stronger efforts should be made to enhance the role of small farms in achieving Mexican food sovereignty. This will not only have benefits in terms of food supply but may also have a wide range of social and environmental benefits.

Keywords: Agricultural supply; small farms; Mexico; farming practices; peasants;

1. Introduction

Attention to the contribution of small farmers to global food supply and to agricultural sustainability is increasing (UNCTAD, 2015; Wiggins et al, 2010; World Bank, 2007; IFAD and UNEP, 2013). Small farms have been associated with beneficial agricultural practices such as seed saving (McGuire and Sperling, 2016), with an agricultural production that relies on little to no external inputs, and with generating livelihoods and reducing poverty (Wiggins et al, 2010, de

Janvry & Sadoulet, 2022). These characteristics of small farms imply that they contribute to the environmental sustainability of the global food system (IFAD and UNEP, 2013; UNCTAD, 2015) and to food sovereignty (Altieri et al., 2012).

Small farms represent 90% of all farms worldwide and provide direct or indirect livelihoods to up to 2.5 billion people (UNCTAD, 2015). Yet, their contribution to the global food supply remains contested. Many documents have claimed that small farms produce 70% to 80% of the global food supply (though this claim is often unsupported by empirical evidence) (Ricciardi et al, 2018). For instance, a recent report states that small farms "provide over 80 per cent of the food consumed in a large part of the developing world" and "the bulk of food in developing countries" (IFAD and UNEP, 2013, pp.6,10). The report cites two sources of evidence to support these claims, one of which is an unpublished speech to the World Agricultural Heritage Forum by its founder and president (also former FAO senior employee) (Koohafkan, 2011). The other is an opinion piece by the president of the International Fund for Agricultural Development (IFAD, 2011), where the same figure is stated without any references. This statement can therefore not be traced back to any evidence or empirical study that can support this claim.

In the face of this unsupported claim that small farms produce most of the global food supply, recent studies have used robust datasets and analytical methods to provide estimates of the share of small farms' production in food supply globally and in different regions. For Ricciardi et al., (2018), small farms (<2ha) produce approximately one third of the global food supply. A similar figure is found in a recent FAO report, which specifies that small farms (<2ha) produce 36% of the world's food (Lowder et al., 2019). Unsurprisingly, these estimates also vary regionally, and depending on the definition that is used to characterise small farms: for Herrero et al. (2017), very small farms (<2ha) contribute to about 30% of the supply of "most food commodities" in Sub-Saharan Africa, Southeast Asia, and South Asia. Samberg et al. (2016, p.1), on the other hand, argue that small farms (<5ha) in Latin America, sub-Saharan Africa, and South and East Asia "are

responsible for more than half of the food calories produced globally, as well as more than half of global production of several major food crops”. A common conclusion of these studies is that further research is needed to quantify the role of small farms to the food supply of different regions.

To partially address this research gap, in this study, we quantify the contribution of small farms to Mexico’s food supply. Mexico has a large diversity of farms in terms of size, socioeconomic, cultural, technological, and production characteristics (FAO, 2018; de Ita, 2014; Schejtman, 1982; Yunez et al., 2013). Mexican studies on rurality have traditionally distinguished two large categories of farms: peasants and business farms (Schejtman, 1982; Toledo et al., 2002; de Ita, 2014). Peasants (or small farmers) are usually defined as those that farm less than 2 ha or 5 ha of cropland, and as having specific characteristics such as consuming part of their production, relying on family labour, using few or no external inputs (such as inorganic fertilisers or pesticides), and having a low income (Schejtman, 1982; Toledo et al., 2002, de Ita, 2014).

The reality of Mexican farms is more complex than this categorisation suggests: a large spectrum of farms exists beyond peasants and business farms, and criteria for categorizing farms vary according to different authors. Schetjman (1982) defined four categories of farms: peasants (relying on family labour), transition farmers (relying on both family and salaried labour, but mainly the second), agricultural entrepreneurs (only salaried labour), and livestock companies (only animal production). For peasant farms he distinguished four categories: infra subsistence (< 4 ha), subsistence (4 - 8 ha), stationary (8-12 ha), excedentary (> 12 ha). For agricultural entrepreneurs he defined three types: small (500 - 1250 labour days per year) medium (1250 - 2500 labour days per year) and large (> 2500 labour days). For livestock companies the types were: small (< 50 cattle heads or equivalent), medium (50 - 300 cattle heads or equivalent), and large (>300 cattle heads or equivalent). Toledo et al (2002) used a socioecological analysis to define an index composed of nine indicators to describe a farm, or any territorial unit in terms of

agriculture type. The typology moves along a scale that goes from peasant to agroindustrial agriculture. The nine criteria used in their analysis are: energy, scale, self-sufficiency, labour (family vs salaried), diversity of crops, labour productivity, energy productivity, type of knowledge used, and cosmovision. For Toledo et al. (2022), farms practising rainfed agriculture on less than 5 hectares are peasant and larger farms or with irrigation no matter the size are agroindustrial. For Yunez et al. (2013), a family farm is one that uses more than 50% of family labour. They also separate specialised family farms, when more than 50% of the income comes from farming, and pluriactivity family farms, when farming income is less than 50%. For de Ita (2014), the two criteria for farm classification are type of labour and size. Peasant farms only use family labour and the size is less than 20 ha. Entrepreneur farms use salary labour and are larger than 20 ha. As we can see, criteria can vary in all these typologies.

Diverse benefits of Mexican small farms have been documented in the literature. Firstly, they contribute to national food security (Galeana-Pizaña et al, 2021) and provide 60% of agricultural labour (FAO, 2018). Yet, quantifications of the contribution of small farms to the domestic supply are lacking. The Mexican government estimates that small farms (<5ha) produce 40% of the country's food supply (SENASICA, 2021), though they do not explain the methodology used to calculate this figure. Secondly, small farms contribute to agrobiodiversity, including genetic and food diversity, as well as associated cultural diversity (de Ita, 2014; Yunez et al., 2013; FAO 2018, Astier et al., 2021). Because they tend to maintain traditional agroecological practices, small farms are thought to be more resource efficient and hence more environmentally sustainable than larger farms (Bustamante et al., 2017).

Mexico's agrarian reform following the Mexican revolution in 1910 distributed almost half of the country's land as communal land (ejidos) to small farmers (Sanchez, 2003). With this, land tenure was recognized to indigenous communities resulting in (1) peasant's food production as the basis of the national production, and (2) preserving traditional practices (Sánchez, 2003), unlike other

Latin American countries which did not have extended agrarian reform and are characterised by unequal land distribution (Kay, 1999). However, this tradition of peasant farming and small farms has been displaced, to a certain extent, by the green revolution of the second half of the twentieth century (de Gortari Rabiela, 2020) and more recently by the impacts of the North American Free Trade Agreement (González, 2006). Since the 1990's, Mexican neoliberal reforms have focused on supporting large-scale and agroindustrial agriculture, thus displacing small farmers (de Ita, 2014). The federal government has recently started to revert this trend, with governmental programs supporting small farmers with technical and financial resources as a double strategy to reach national food sovereignty and to alleviate rural poverty (DOF, 2020; SADER, 2017). This strategy could be enhanced by robust and recent studies that analyze the characteristics of small farms, including their agricultural practices and the share of their production in the Mexican food supply to discuss their potential role, and changes needed, to reach Mexican food sovereignty.

The aim of this study is to quantify how much of the agricultural supply in Mexico is produced by small farms, and to analyse the farming practices used to produce it. To do this, we answer the following research questions: (1) How much of the agricultural food products are produced in Mexico by farms of different sizes? (2) What farming practices are used by farms of different sizes? (3) How much of the agricultural supply is produced by small farmers? With this, we discuss the role of small farmers for food sovereignty, and some implications of their use of different farming practices. The insights of this paper can be used to design agricultural programs to support small farmers as a means to reach national food sovereignty.

2. Material and methods

System description and analytical steps

The system of this paper is composed of two elements (agricultural production and agricultural supply), which are analysed in three separate steps: type of farm, farming practices, and origin of supply (Figure 1).

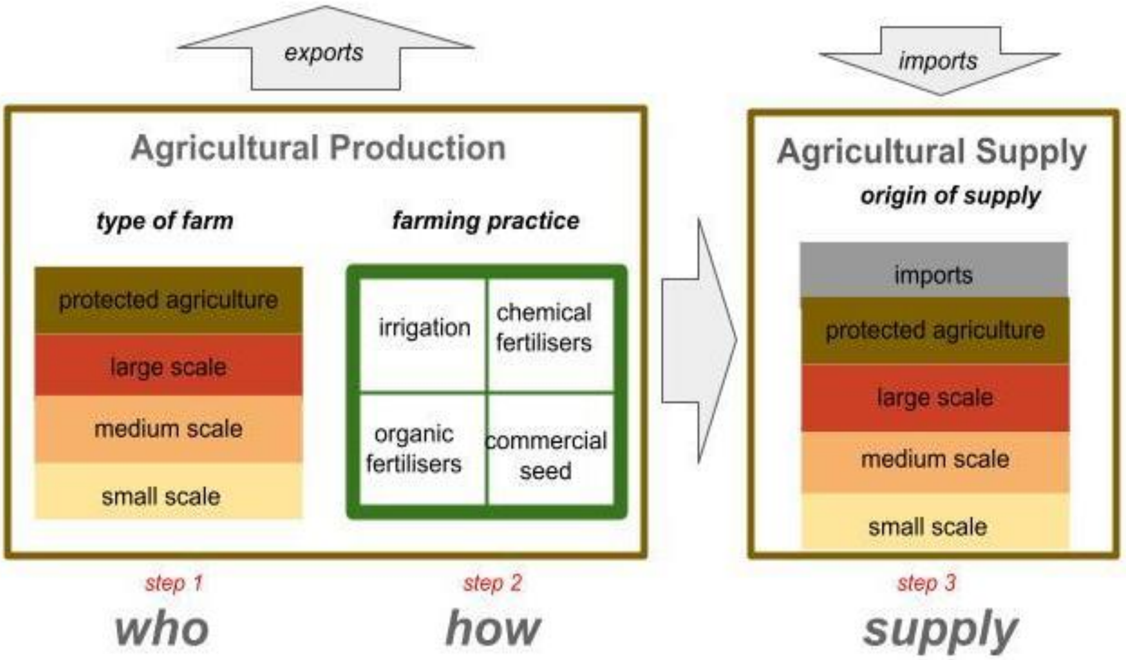

Figure 1. System description. Figure designed by the authors.

Table 1. Criteria for grouping the farms by type. The criteria are based on the sized-based strata defined by the survey (INEGI, 2019a).

Type of farm	***Criteria for each type of agricultural products***			
	**Crops**	**Cattle products**	**pig products**	**poultry products**
**Small scale**	5 ha or less	≤ 26 animals	≤16 animals	≤ 500 animals
**Middle scale**	5 ha - 50 ha	27-120 animals	17-150 animals	500-20,000 animals
**Large scale**	> 50 ha	> 120 animals	>more than 150 animals	> 20,000 animals
**Protected agriculture**	Farms that use protection technologies (microtunnels or greenhouses) to produce crops.			

NOTE: The survey groups cropland farms in six strata based on their cropland area, and livestock farms in four strata based on their number of animals. For crop farms, large-scale farms include strata 1 (50ha>x) and strata 2 (50ha> x <20ha); medium-scale farms include strata 3 (20ha> x <10ha) and strata 4 (10ha> x <5ha); and small-scale farms include strata 5 (5ha> x <2ha); and strata 6 (x<2). For cattle and pig farms, large-scale farms include strata 1 (120 cows >x & 150 pigs >x); mediums-scale farms include strata 2 (120 cows > x < 36 cows & 150 pigs> x <16 pigs); and small-scale farms include strata 3 (36 cows> x < 11 cows & 16 pigs> x < 3 pigs) and strata 4 (x<11 cows & x<2 pigs).

The Mexican agricultural production is the starting point of the analysis. It is defined as the total amount of agricultural products (crops and animal products) that Mexican farms produce (step 1). The criteria to categorise the type of farm are the area of cropland and the number of animals (Table 1), since these are the most common criteria in the literature (Chalate-Molina et al., 2010;

Herrero et al. 2017; de Ita, 2014; Lowder et al., 2019; Ricciardi et al., 2018; Samberg et al. 2016; Schejtman, 1987). Protected agriculture is considered as a different category because, compared with non-protected agriculture, its production is mainly determined by investment, technology level, and knowledge of farmers about greenhouse operation (Moreno Reséndez et al., 2011; Vargas Canales et al., 2015), as well as because it can be practiced in areas unsuitable for open agriculture (Moreno Reséndez et al., 2011). In addition, protected agriculture is more efficient in terms of resource use because of the use of intensive production systems with high yielding seeds and because the environmental factors can be controlled, thus reducing risks of losses and enabling crop production all year round (Jensen & Malter, 1995).

Then, we analysed the farming practices used by Mexican farms (step 2). Four indicators were used to define the farming practices: use of irrigation, use of inorganic fertilizers, use of organic fertilizers, and use of commercial seeds.

Finally, we calculated how much of the national agricultural supply (including imports and excluding exports) is produced by small farmers in Mexico (step 3). We use the concept of domestic supply defined by the FAO,[1] which includes all primary production available in the country as well as the losses in the food chain, to define the national agricultural supply (figure 1).

Materials: Data source

The data source is the 2019 Mexican National Survey of Agriculture, produced periodically by the Mexican federal government (INEGI, 2019a). We use the microdata of the survey (a database

[1] The FAO definition of domestic supply includes the primary commodities produced in the country, minus exports, plus imports, plus changes in stock (FAO, 2001). For this study, the changes in stock are not considered. Note that the domestic supply includes food products such as agricultural products (crops and animal products) and processed foods (e.g. sugars, alcoholic beverages, and vegetable oils), and also non food products (e.g. crop's products for pharmaceutics & textiles), feed products, commodities for processed production, and losses in the food chain.

including the individual responses of each respondent). This dataset is not publicly available to ensure confidentiality for the respondents. Access to process and analyse the microdata was granted by the National Institute of Statistics and Geography (INEGI) through its Laboratory of Microdata for research purposes.

The survey presents data for the 29 major agricultural products (Table 2). These 29 products, which are those considered in this study, account for 87% of the Mexican agricultural production and 84% of the Mexican domestic supply in 2019 (Appendix 1, data from FAO (2022a, 2022b, 2022c)).

Table 2. Agricultural products analysed in this study (categorised by food group). Source of data: authors' categorisation based on INEGI (2019a).

**Food group**	**Agricultural products**
white maize	white maize
other cereals	wheat, rice, amaranth
bean	bean
vegetables	squash, onion, chilli pepper, tomato
fruits	avocado, strawberry, lemon, mango, apple, orange, banana, grape
animal feed	yellow maize, alfalfa, sorghum, soybean
animal products	milk, cattle meat, pork, chicken meat, eggs
stimulants	cocoa, coffee
sugar cane	sugar cane

The survey was applied to 63,855 farms, which is a statistically representative sample for each of the food products and types of farms. Sampling weights were included in the original database (INEGI, 2019b). In the survey, farms are categorized in six strata (crop farms) and four strata (livestock farms) based on their amount of cropland area and livestock per farm (INEGI, 2019a), see Table 1. The survey does not assign strata for poultry production; therefore the selection was based on expert knowledge (González-Cerón, 2022).

Methods: Data analysis

Crop farms

For farms that produce crops, each respondent reports the production and cultivated area per crop , taking into account crops intended to be sold on the market, those kept as seeds for the next season, those used for feed, and those intended for home consumption. Farms with open agriculture (unlike those practising protected agriculture) report the share of their production that is dedicated to each use; this data is presented in Appendix 2.

Using the sampling weights, these values are aggregated by crop and type of farm using the *"collapse (sum)"* function of STATA software. The results are shown in Table 3.

Livestock farms

As part of the survey, each respondent reported the number of animals on the farm and its production purpose (e.g. beef cows, laying hens, milking cows). We used this data, and its associated sampling weights to estimate the annual animal products production in each type of farm as follows.

The total number of animals per type of farm was calculated with the *"collapse (sum)"* function of STATA software by aggregating each type of animal reported by type of farm using the sampling weights. Having the total animals in each type of farm, we estimated the share of animals in each farm (e.g. x% of national milking cows are in small-scale farms). This percentage was multiplied by the national annual production of beef, milk, pork, chicken meat, and eggs, respectively, reported by the Agriculture and Fishery Information System of Mexico in 2019 (SIAP, 2022). With this, we estimated the amount of each animal product produced by each type of farm. The results are shown in Table 3.

For beef production, we included beef cows and half of the "dual" animals (milk and beef producing cows). For milk production, we included the milking cows and half of the "dual"

animals. For pig production, we considered all pigs reported by each farm (any age). The sample for poultry production farms is a deterministic sample which does not come with sampling weights, which means that the total national values cannot be quantified. To address this, we used the distribution of these farms among the small-, medium-, and large- scale farms to estimate the national distribution of these farms. The sample of the poultry production farms represent 90% of the national production of eggs and chicken meat (source: personal communication with the staff of the National Agricultural Surveys of INEGI). To estimate the poultry meat production, we considered growing chickens, and for egg production, we considered the hens reported by each farm.

Farming practices

Each respondent reported whether they used an irrigated or rainfed system, applied inorganic fertilizers and/or organic fertilizers, and the type of seed used. The organic fertilizers used by farmers are mainly manure of different livestock, compost, and other homemade fertilizers such as earthworm humus (INEGI, 2019a). For the type of seed, respondents could choose from four categories: (1) landrace or farm saved seeds, (2) improved (hybrid varieties or varieties that have been selected by a rigorous methods to assure quality, performance and yield if environmental conditions are optimal), (3) certified or registered, (4) genetically modified. We grouped the improved, certified or registered, and the genetically modified seeds as "commercial seeds", and the landrace or farm saved seeds as "non commercial seeds". These data were used to define four variables characterizing farming practices: (a) use of irrigation, (b) use of inorganic fertilizers, (c) use of organic fertilizers, (d) use of commercial seeds. Farming practices of protected agriculture are not reported in the survey, so these farms were not considered in the analysis. This analysis also excluded animal products; but by including fodder crops, we considered these products indirectly. The answers of all respondents were aggregated per type of farm and type of farming practice, using sampling weights, with the *"collapse (sum)"* function of STATA software. The results are shown in Figure 2.

Agricultural supply

Finally, to calculate the agricultural supply, we quantified the exports and imports of the 29 agricultural products included in this study (Appendix 3). The exports were subtracted equally for all types of farms from the national production, and the imports were added as a new category making up the domestic supply (Figure 1, step 3). With this, we calculated how much of the agricultural supply is produced by small farms, farms of other sizes, or obtained from imports. See Appendix 3 for details.

3. Results

3.1 Agricultural production in Mexico by type of farm

Most of the food produced in Mexico (64%) comes from large farms (Table 3). Small farms produce 19%, medium-scale farms produce 33%, and protected agriculture produce 2% of the Mexican agricultural production. The share of the production of each type of agricultural product is also different among the four types of farms (Appendix 4). Almost half of the white maize (the main food product in the Mexican diet) is produced by large-scale farms, and 17% is produced by small farms. Beans, also one of the main food products of the Mexican diet, are mainly produced by large-scale farms, followed by medium farms, while small farms produce 6% of the national production. Vegetables are mainly produced by large-scale farms, followed by protected agriculture farms (the latter producing almost exclusively vegetables). Animal products and fodder crops are also mostly produced by large-scale farms (68% and 62%), followed by medium-scale farms (18% and 27%), and by small-scale farms (14% and 16%).

Even though small-scale farms produce a relatively small share of the national production, they account for the largest share of farms (57% of crop farms, 78% of cattle farms, 89% of pig farms, and 96% of poultry farms) (Table 3). This difference in the number of farms of different sizes and their total production can be explained by the fact that the average production per farm is much

higher in large scale farms, followed by medium- and small- scale farms (see 4th row in Table 3). Large-scale farms' average production is ten times higher than small-scale farms for crops, 70 times higher for cattle farms, and 2000 times higher for pig farms. However, the average crop yield is similar among the three types of farms (Table 3). Protected agriculture, on the other hand, boasts yields ten times higher than all other farms - this can be explained by the fact that it produces almost exclusively vegetables, which yields are higher than for other types of crops (such as cereals).

Table 3. Distribution of farms, production, and cropland among the type of farms, and productivity indicators. Source: calculations by the authors using INEGI (2019a). See Appendix 4 for data by food item and Supplementary Information for details

		Small scale		Medium scale		Large scale		Protected agriculture		Total	
Number of Farms [Million farms]	Crops	1.97	*57%*	1.05	*30%*	0.43	*12%*	0.02	1%	3.46	*100%*
	Cattle	1.09	*78%*	0.27	*19%*	0.04	*3%*	N.A.	N.A.	1.40	*100%*
	Pig	0.34	*17%*	0.04	*2%*	1.60	*81%*	N.A.	N.A.	1.98	*100%*
	Poultry	N.A.	*96%*	N.A.	*0%*	N.A.	*4%*	N.A.	N.A.	N.A.	*100%*
Production [Million tonnes]	Crops	23.27	*20%*	41.77	*36%*	48.86	*42%*	2.63	*2%*	116.53	*100%*
	Cattle	3.09	*21%*	3.84	*26%*	7.59	*52%*	N.A.	N.A.	14.52	*100%*
	Pig	0.13	*8%*	0.13	*8%*	1.34	*84%*	N.A.	N.A.	1.60	*100%*
	Poultry	0.00	*0.0%*	0.00	*0.1%*	6.42	*99.9%*	N.A.	N.A.	6.43	*100%*
Cropland area [Mha]	Crops	3.27	*21%*	5.15	*33%*	7.24	*46%*	0.05	*0.3%*	15.71	*100%*
**Average farm Production [ton / farm]**	**Crops**	**11.83**		**39.89**		**114.61**		**145.69**		**33.69**	
	**Cattle**	**2.83**		**14.43**		**199.59**		**N.A.**		**10.38**	
	**Pig**	**0.39**		**3.36**		**0.84**		**N.A.**		**0.81**	
	**Poultry**	**N.A.**		**N.A.**		**N.A.**		**N.A.**		**N.A.**	
**Average yield [ton/ha]**	**Crops**	**7.12**		**8.11**		**6.75**		**50.86**		**7.42**	
	**Cattle**	**N.A.**		**N.A.**		**N.A.**		**N.A.**		**N.A.**	
	**Pig**	**N.A.**		**N.A.**		**N.A.**		**N.A.**		**N.A.**	
	**Poultry**	**N.A.**		**N.A.**		**N.A.**		**N.A.**		**N.A.**	

The production values presented in Table 3 include products that are sold by farmers as well as those intended for other uses (animal feed, seeds and home consumption). Only 6%, 4% and 9 % of the production is not sold by farmers, for small-, medium and large-scale farms respectively (Appendix 4). This percentage diverges among food crops. For instance, less than 90% of maize, beans, and alfalfa are sold - across all farm types. This means that the tree type of farms use a similar share of their production of these crops for food, feed and seeds.

3.2 Farming practices used in the production

Farming practices are similar across the three types of farms (Figure 2). Over 90% of farms of any size use inorganic fertilizers. Less than a quarter of farms (of any size) use organic fertilizers. This means that most crops are produced with inorganic fertilizers and without organic fertilizers across all farm sizes. The use of irrigation systems shows a different pattern. It is mostly used by large-scale farms (66%) but is also commonly used by small- and medium-scale farms (53% and 47% respectively).

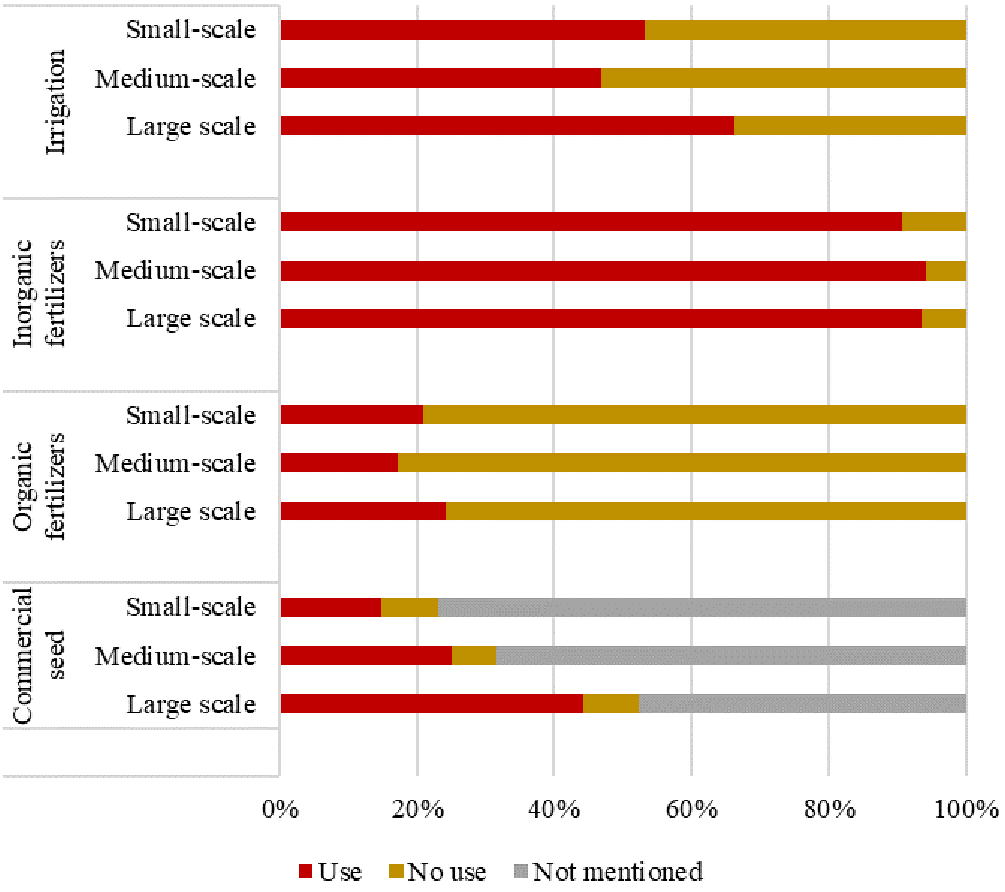

Figure 2. Farming practices used in the production of crops per type of farm. Source of data: calculations by the authors using the National Agricultural Survey (INEGI, 2019a). The data plotted in this graph can be found in the Supplementary Information.

Most farms failed to report the type of seeds they use (see grey values in Figure 2). Only 23%, 32% and 52% of the small-, medium, and large-scale farms, respectively, reported the type of

seed used. Therefore, more data is needed to discuss the distribution of the type of seeds among small-, medium-, and large scale farms.

3.3 How much of the agricultural supply is produced by small farms?

7.8 million tons out of the 139 million tons of the agricultural products produced in Mexico are exported, which accounts for 6% of the national production (Appendix 3, Table A6). These are mainly vegetables (2.6 million tonnes) and fruits (2.2 million tonnes). The imports of the agricultural products considered in this study represent 36.95 million tons per year and are mainly animal feed (26.8 million tons: mainly yellow maize) and other cereals for food (mainly wheat which amounts to 5.5 million tons). By adding the imports and subtracting the exports from the national agricultural production, we obtain the national supply of agricultural products, which amounts to 168 million tons in 2019. 22% of the supply originates from imports, 15% is produced by Mexican small-scale farms, 26% by Mexican medium-scale farms, 36% by large-scale farms, and only 1% by protected agriculture (Figure 3, Table 4, Appendix 3).

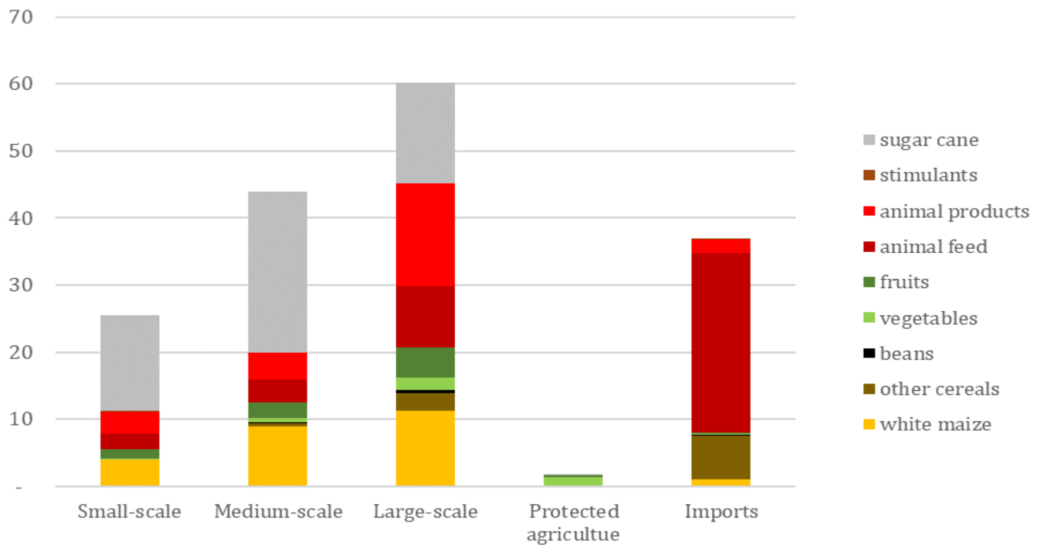

Figure 3. Source of the Mexican domestic supply in million tons, by food group. Source of data: calculations by the authors using the National Agricultural Survey (INEGI, 2019a) and FAO (2022b). The data plotted in this graph can be found in Table A5, Appendix 4, and in the Supplementary Information.

Table 4. Source of national agricultural supply for each food product million tons and share of the total supply by food product (in italics). Source of data: calculations by the authors using the National Agricultural Survey (INEGI, 2019a) and FAO (2022a). The details on the values with decimal points are shown in the Supplementary Information.

Food group	Agricultural Product	Small scale	Medium scale	Large scale	Protected agriculture	Imports	Total
white maize	white maize grain	3.99	9.02	11.23	0.00	1.12	25.36
		16%	*36%*	*44%*	*0%*	*4%*	*100%*
other cereals	wheat	0.02	0.29	2.59	-	5.53	8.42
		0.2%	*3%*	*31%*	*0%*	*66%*	*100%*
	rice	0.01	0.04	0.10	-	0.94	1.08
		1%	*3%*	*10%*	*0%*	*86%*	*100%*
	amaranth	0.004	0.001	0.001	0.000	-	0.01
		68%	*20%*	*11%*	*1%*	*0%*	*100%*
	**TOTAL**	**0.03**	**0.32**	**2.69**	**0.00**	**6.47**	**9.51**
		0%	***4%***	***28%***	***0%***	***68%***	***100%***
bean	bean	0.05	0.25	0.48	0.00	0.12	0.90
		5%	*28%*	*54%*	*0%*	*13%*	*100%*
vegetables	squash	0.02	0.03	0.08	0.00	-	0.13
		17%	*20%*	*62%*	*1%*	*0%*	*100%*
	onion	0.03	0.10	0.63	0.00	0.09	0.85
		4%	*12%*	*74%*	*0%*	*11%*	*100%*
	chilli pepper	0.03	0.41	0.54	0.36	0.00	1.34
		3%	*30%*	*40%*	*27%*	*0%*	*100%*
	tomato	0.03	0.07	0.51	1.01	0.00	1.62
		2%	*4%*	*31%*	*63%*	*0%*	*100%*
	**TOTAL**	**0.12**	**0.60**	**1.75**	**1.37**	**0.09**	**3.94**
		3%	***15%***	***45%***	***35%***	***2%***	***100%***
fruits	avocado	0.22	0.41	0.37	-	-	1.01
		22%	*41%*	*37%*	*0%*	*0%*	*100%*
	strawberry	0.01	0.01	0.02	0.16	0.00	0.20
		4%	*6%*	*9%*	*79%*	*2%*	*100%*
	lemon	0.09	0.22	0.65	0.00	0.00	0.97
		10%	*23%*	*67%*	*0%*	*0%*	*100%*
	mango	0.15	0.21	0.39	0.00	0.00	0.76
		20%	*28%*	*52%*	*0%*	*0%*	*100%*
	apple	0.02	0.15	0.22	0.09	0.16	0.64
		3%	*23%*	*35%*	*14%*	*25%*	*100%*
	orange	0.64	1.22	1.60	0.00	0.02	3.48
		18%	*35%*	*46%*	*0%*	*1%*	*100%*
	banana	0.22	0.11	1.14	0.00	0.00	1.47
		15%	*7%*	*78%*	*0%*	*0%*	*100%*
	grape	0.01	0.01	0.15	0.00	0.06	0.23
		3%	*5%*	*65%*	*1%*	*26%*	*100%*
	**TOTAL**	**1.36**	**2.34**	**4.55**	**0.25**	**0.25**	**8.75**
		15%	***27%***	***52%***	***3%***	***3%***	***100%***

Table 4. *continued.*

Food group	Agricultural Product	Small scale	Medium scale	Large scale	Protected agriculture	Imports	Total
animal feed	yellow maize grain	0.43	0.93	3.98	-	19.70	25.05
		2%	*4%*	*16%*	*0%*	*78%*	*100%*
	alfalfa	1.55	1.50	2.56	0.00	-	5.61
		28%	*27%*	*45%*	*0%*	*0%*	*100%*
	sorghum grain	0.35	0.93	2.39	-	0.63	4.30
		8%	*21%*	*56%*	*0%*	*15%*	*100%*
	soybeans	0.00	0.03	0.27	0.00	6.48	6.78
		0%	*1%*	*4%*	*0%*	*95%*	*100%*
	**TOTAL**	**2.34**	**3.39**	**9.20**	**0.00**	**26.80**	**41.74**
		**6%**	**8%**	**22%**	**0%**	**64%**	***100%***
animal products	milk	2.57	3.28	6.64	-	0.21	12.70
		20%	*26%*	*52%*	*0%*	*2%*	*100%*
	beef	0.50	0.53	0.90	-	0.00	1.93
		26%	27%	47%	0%	0%	*100%*
	pork	0.13	0.13	1.34	-	0.70	2.30
		6%	*5%*	*58%*	*0%*	*31%*	*100%*
	chicken meat	0.00	0.00	3.47	-	0.97	4.45
		0%	*0%*	*78%*	*0%*	*22%*	*100%*
	eggs	0.00	0.00	2.95	-	0.05	3.00
		0%	*0%*	*98%*	*0%*	*2%*	*100%*
	**TOTAL**	**3.20**	**3.94**	**15.30**	**-**	**1.94**	**24.38**
		13%	***16%***	***63%***	***0%***	***8%***	***100%***
stimulants	cocoa	0.01	0.01	0.00	-	0.04	0.06
		23%	*10%*	*5%*	*0%*	*62%*	*100%*
	coffee	0.09	0.06	0.04	0.00	0.12	0.31
		29%	*20%*	*13%*	*1%*	*37%*	*100%*
	**TOTAL**	**0.11**	**0.07**	**0.05**	**0.00**	**0.16**	**0.38**
		28%	***18%***	***12%***	***1%***	***41%***	***100%***
sugar cane		14.35	24.00	14.90	0.00	-	53.25
		27%	*45%*	*28%*	*0%*	*0%*	*100%*
**Total agricultural supply**		**25.56**	**43.94**	**60.15**	**1.63**	**36.95**	**168.22**
		15%	***26%***	***36%***	***1%***	***22%***	***100%***

Different food products are sourced in different ways (Table 4). For instance, wheat, rice, animal feed, and cocoa are the food categories where imports represent more than half of the domestic supply. For these categories, the difference between the production of small-, medium-, and large-scale farms appears to be relatively less important when imports are considered. Only 0.2%, 1%, and 6% of the domestic supply of wheat, rice, and animal feed, respectively, is produced by small farmers.

The food products that are most largely produced by small farms (at least 20% of the domestic supply), in comparison with the other farms or imports, are amaranth (68%), avocado (22%), mango (20%), alfalfa (28%), milk (20%), beef (26%), sugar cane (27%), cocoa (22%), and coffee (29%). Al these products are considered "cash crops" or "commercial crops", animal products or animal feed. None of them are staple foods. Also, production by small-scale farms never exceeds 30% of the national supply, apart from amaranth, but its production accounts to only 5.4 thousand tonnes in 2019, which is only 0.003 % of the total agricultural supply.

4. Discussion and conclusion

Mexican small farms, characterised in our study as those that have less than 5 hectares of cropland, 16 pigs or less, 26 cows or less, or 500 chickens or less, produce 19% of Mexico's agricultural production, and 15% of Mexico's agricultural supply. The reduction in the percentage from production to supply is explained by the large quantities of food imports. These values differ from the widely reported statement that small farmers produce most of the food globally, and the Mexican government's statement that small farmers produce 40% of the Mexican food (SENASICA, 2021). Recent studies (Ricciardi 2018 et al.; Samberg et al., 2016) estimating the global production of small farms have already shown a smaller estimate than the unsupported figure of 70%-80%. These authors estimate that small farms (under 5 hectares) produce 44%-48% (Ricciardi et al., 2018) and 55% (Samberg et al., 2016) of global food supply. Still, their results are much higher than the estimates of this study. This can partly be explained by the fact that small farms are more common to Africa and Asia than to Latin America (Samberg et al., 2016). This can also illustrate how Mexican small farms and the peasant farming traditions which had been preserved thanks to the agrarian reform of the early 20th century are losing importance as a result of the wave of neoliberal reforms of the last three decades (de Ita, 2014).

Similarly to Ricciardi et al (2018), our results show that farms of different sizes tend to produce different food products. Ricciardi et al. show that small farms produce mainly staple food crops (cereals, roots and tubers, pulses) and fruits, and large scale farms produce oil crops, vegetables, and other crops. However, we found a different pattern for the Mexican case. The main staple food crops for Mexican diets (white maize and beans) are mainly produced by large-scale farms. In contrast, the agricultural products that small farms most produce are "commercial" or "cash crops": coffee and cocoa, sugar cane, followed by animal products, fruits, animal feed, and white maize; with only 16% of white maize being produced by small-scale farms (Table 4).

Further, farming practices do not differ widely according to the farm size (Figure 2). The farming practices of small-, medium-, and large- scale farms follow a similar pattern: most of the food is produced using inorganic fertilizers, around half of farms of any size use irrigation, and a low share of the production relies on organic fertilizers. However, these findings must be corroborated and expanded through further research. In this analysis, we only considered a dichotomous characterisation of farming practices (whether farms use a certain agricultural input or not). But differences may exist in the amount or degree of use among the farms. For instance, small farms may use a smaller amount of fertilizer and irrigation than large scale farms. These differences must be quantified in a further study. In addition, the use of non-commercial seed has several social, cultural and agrobiodiversity implications. Efforts have been made to preserve diversity in Mexican seeds, particularly for native maize varieties which are preserved mostly by small farmers (Ortega, 2021). Unfortunately, as most farms fail to report the type of seed they use, our study does not allow us to draw conclusions regarding the prevalence of the use of commercial seeds in Mexican farms. Further research is hence needed to identify the type of seeds used in Mexican agricultural production to enable discussions on the related agrobiodiversity, cultural and political implications of the use of different seeds.

Thus, our results show that Mexican small farms are not the main contributors to the Mexican agricultural supply, and their farming practices may have similar environmental implications than medium- and large- scale farms. However, these findings do not diminish the relevance of small farms for Mexico's food sovereignty and employment. Small farms represent the majority of farms in Mexico, a fifth of the cropland area, and the main provider of rural livelihoods. Small farms use marginal lands which otherwise would not be used by large-scale farms. In addition, they are a space for the generation of traditional knowledge and cultural heritage, which is a key tool for the resilience and adaptability of the national food system (Altieri & Nicholls, 2017; Altieri, 2004). Recently, some efforts have been made by the Mexican government to support small farmers (DOF, 2020), however they have not been enough. Since the 1990's, the governmental agricultural programs for small farmers have focused on increasing family income, rather than on enhancing production capacity (Yunez et al., 2013). The fact that small farms persist with little governmental support suggests that their production system is resilient due to their social land tenure (de Ita, 2014), to their culture and cosmovision (Bartra 2020; Leff 2020; Toledo 2002), and to their economic strategies (Bartra 2020). According to some authors (de Janvry & Sadoulet, 2022; Wiggins et al. 2010) small farms have the potential to increase their food productivity increased while reducing poverty.

To increase the relevance of small farms in Mexican food sovereignty, stronger efforts are needed to increase their role in the production of the main staple and nutritious food for the Mexican population, which will not only have benefits in terms of enhancing food sovereignty, but also may have a wide range of social and environmental benefits. This can be the focus of further agricultural programs promoting the production of nutritious staple food such as beans and white maize.

Conflict of Interests: The authors declared that they have no conflict of interest.

References

Altieri, M. A. (2004). Linking ecologists and traditional farmers in the search for sustainable agriculture. Frontiers in Ecology and the Environment, 2(1), 35-42. https://doi.org/10.1890/1540-9295(2004)002[0035:LEATFI]2.0.CO;2

Altieri, M. A., Funes-Monzote, F. R., & Petersen, P. (2012). Agroecologically efficient agricultural systems for smallholder farmers: contributions to food sovereignty. Agronomy for sustainable development, 32(1), 1-13. https://doi.org/10.1007/s13593-011-0065-6

Altieri, M. A., & Nicholls, C. I. (2017). The adaptation and mitigation potential of traditional agriculture in a changing climate. Climatic Change, 140(1), 33-45. https://doi.org/10.1007/s10584-013-0909-y

Astier, M., Perales Rivera, H., Orozco Ramírez, Q., Aragón Cuevas, F., Bye, R., Linares, E. y Mera Ovando, L. M. (2021). Conservación de la agrobiodiversidad en México: propuestas y experiencias en el campo. Comisión Nacional para el Conocimiento y Uso de la Biodiversidad (conabio) / Comisión Nacional de Áreas Naturales Protegidas (conanp). Ciudad de México. 212 pp. https://www.researchgate.net/publication/353851339_Conservacion_de_la_agrobiodiversidad_en_Mexico_Propuestas_y_experiencias_en_el_campo

Bartra, A. (2020) Repensar lo rústico, aportes a una teoría del campesinado contemporáneo. En: Boltvinik, J y Mann, S A. (Coords). Pobreza y persistencia campesina en el siglo XXI. pp 113-133. CROP, Editorial: Siglo Veintiuno Editores: México.

Bustamante, T., Carrera, B., & Schwentesius Rindermann, R. (2017). Sostenibilidad de pequeños productores en los Tianguis Orgánico de Tlaxcala, Puebla y Oaxaca, Veracruz. Estudios Regionales en Economía, Población y Región, 37(1), 1-33. https://doi.org/10.20983/epd.2017.37.1

Chalate-Molina, H., Gallardo-López, F., Pérez-Hernández, P., Paul Lang-Ovalle, F., Ortega-Jiménez, E., & Vilaboa Arroniz, J. (2010). Características del sistema de producción bovinos de doble propósito en el estado de Morelos, México. Zootecnia tropical, 28(3), 329-339. http://ve.scielo.org/scielo.php?script=sci_arttext&pid=S0798-72692010000300004

DOF, (2020) PROGRAMA Sectorial de Agricultura y Desarrollo Rural 2020-2024 (Sectoral Program for agriculture and rural development 2020-2024, Mexico). Federation.
Diario Oficial de la Federación (Official Journal of the Mexican Federation). Last access: December 2022, from: https://dof.gob.mx/nota_detalle.php?codigo=5595549&fecha=25/06/2020

FAO, (2018) El México rural del siglo XXI. FAO. México. Last access: December 2022, from: https://www.fao.org/3/i9548es/I9548ES.pdf

FAO (2001) Concepts and definitions used In Food Balance Sheets. Food Balance Sheets, a Handbook. Food and Agricultural Organization of the United Nations, Rome 2001. Last access: January 2023, from https://www.fao.org/3/x9892e/X9892e02.htm

FAO, (2022a) Food Balance Sheets of the FAO. FAOSTAT. Last access: December 2022, from: https://www.fao.org/faostat/en/#data/FBS

FAO, (2022b) Detail Trade Matrix of the FAO. FAOSTAT. Last access: December 2022, from: https://www.fao.org/faostat/en/#data/TM

FAO, (2022c) Crops and livestock products. FAOSTAT. Last access: December 2022, from: https://www.fao.org/faostat/en/#data/QCL

Galeana-Pizaña, J. M., Couturier, S., Figueroa, D., & Jiménez, A. D. (2021). Is rural food security primarily associated with smallholder agriculture or with commercial agriculture?: An approach to the case of Mexico using structural equation modelling. Agricultural Systems, 190, 103091. https://doi.org/10.1016/j.agsy.2021.103091

Gonzalez, C. G. (2006). Markets, monocultures, and malnutrition: agricultural trade policy through an environmental justice lens. Michigan State Journal of International Law, 14, 345.

González Cerón, Fernando (2022) Personal communication with Dr. Fernando González Cerón, expert on Mexican poultry production. https://www.researchgate.net/profile/Fernando-Gonzalez-Ceron

de Gortari Rabiela, R. (2020). De la revolución verde a la agricultura sustentable en México. Nueva Antropología, 33(92), 66–86. Recuperado a partir de https://revistas.inah.gob.mx/index.php/nuevaantropologia/article/view/15995

Herrero, M., Thornton, P. K., Power, B., Bogard, J. R., Remans, R., Fritz, S., ... & Havlík, P. (2017) Farming and the geography of nutrient production for human use: a transdisciplinary analysis. The Lancet Planetary Health, 1(1), e33-e42. https://doi.org/10.1016/S2542-5196(17)30007-4

IFAD. 2011. Viewpoint: Smallholders can feed the world. Rome: International Fund for Agricultural Development. Last access: january 2023, From: https://www.ifad.org/documents/38714170/40706188/Smallholders+can+feed+the+world_e.pdf/460ca6c2-7621-40d8-9f79-a56f6f8fa75e

IFAD & UNEP, (2013) "Smallholders, food security, and the environment". Report by the International Fund for Agricultural Development (IFAD) and the United Nations Environment Program (UNEP). Last access: december 2023 from: https://www.ifad.org/documents/38714170/39135645/smallholders_report.pdf/133e8903-0204-4e7d-a780-bca847933f2e

INEGI (2019a) Encuesta Nacional Agropecuaria 2019. Instituto Nacional de Estadística y Geografía (INEGI). Last access: June 2023 from: https://www.inegi.org.mx/programas/ena/2019/

INEGI (2019b) Metodología Encuesta Nacional Agropecuaria 2019. Instituto Nacional de Estadística y Geografía (INEGI). Las access: June 2023 from: https://www.inegi.org.mx/app/biblioteca/ficha.html?upc=702825197315

De Ita, A. (2014). México: Economía campesina y agricultura empresarial, veinte años después. REVISTA ALASRU, 53. Last access: december 2022, from : https://www.ceccam.org/sites/default/files/Revista%20ALASRU.pdf#page=53

de Janvry, A., & Sadoulet, E. (2022). Agriculture for Development: Analytics and Action. Annual Review of Resource Economics, 14. 1-16. https://doi.org/10.1146/annurev-resource-090921-045011

Jensen, M. H., & Malter, A. J. (1995). *Protected agriculture: a global review.* Washington D. C. World Bank. p.p. 157

Kay, C. (1999). Mirando hacia atrás: el tiempo de las reformas agrarias. Revista Envío, (208). https://www.envio.org.ni/articulo/952

Koohafkan, P. (2011) Globally important agricultural heritage systems. Presentation at the International Forum on Globally Important Agricultural Heritage Systems (GIAHS), Beijing, 9-12 June 2011

Leff, E. (2020) De la persistencia del campesinado en el capitalismo al ambientalismo de los pueblos indígenas y la sostenibilidad de la vida. En: Boltvinik, J y Mann, S A. (Coords). Pobreza y persistencia campesina en el siglo XXI. pp 253-271. CROP, Siglo Veintiuno Editores: México.

Lowder, S.K., Sánchez, M.V. & Bertini, R. (2019) Farms, family farms, farmland distribution and farm labour: What do we know today? FAO Agricultural Development Economics Working Paper 19-08. Rome, FAO. Last access: December 2022, from: https://www.fao.org/agrifood-economics/publications/detail/en/c/1252236/

McGuire, S., & Sperling, L. (2016). Seed systems smallholder farmers use. Food Security, 8(1), 179-195. https://doi.org/10.1007/s12571-015-0528-8

Moreno Reséndez, A., Aguilar Durón, J., & Luévano González, A. (2011). Características de la agricultura protegida y su entorno en México. Revista Mexicana de agronegocios, 29(1345-2016-104296), 763-774. https://doi.org/10.22004/ag.econ.114479

Ortega, R. (2021). Estudios de diversidad, conservación in situ y mejoramiento de maíces nativos en México. Universidad Autónoma Chapingo, Texcoco, México.

Ricciardi, V., Ramankutty, N., Mehrabi, Z., Jarvis, L., & Chookolingo, B. (2018). How much of the world's food do smallholders produce?. Global food security, 17, 64-72. https://doi.org/10.1016/j.gfs.2018.05.002

SADER (2017) “Programa Apoyos a Pequeños Productores, campo joven y con raíces” Blog of the Ministry of Agriculture and Rural Development (Secretaría de Agricultura y Desarrollo Rural SADER). Last access: January 2023, from: https://www.gob.mx/agricultura/es/articulos/programa-apoyos-a-pequenos-productores-campo-joven-y-con-raices#:~:text=El%20programa%20de%20Apoyos%20a,peque%C3%B1os%20productores%20con%20servicios%20de

SAGARPA, (2017). Planeación Agrícola Nacional 2017-2030 (National Agricultural Plan 2017-2030). Ministry of Agriculture, Rural Development, Fishery and Food (Secretaría de Agricultura, Ganadería, Desarrollo Rural, Pesca y Alimentación). (SAGARPA)Last Access: January 2023, From: https://www.gob.mx/cms/uploads/attachment/file/255627/Planeaci_n_Agr_cola_Nacional_2017-2030-_parte_uno.pdf

Samberg, L. H., Gerber, J. S., Ramankutty, N., Herrero, M., & West, P. C. (2016). Subnational distribution of average farm size and smallholder contributions to global food production. Environmental Research Letters, 11(12), 124010. http://doi.org/10.1088/1748-9326/11/12/124010

Sánchez, J. C. M. (2003). Reforma agraria: del latifundio al neoliberalismo. Ed. Plaza y Valdés. p.p. 238. ISBN: 970-722-180-1

SENASICA (2021) Blog de: Servicio Nacional de Sanidad, Inocuidad y Calidad Agroalimentaria (SENASICA) *National Service of Health, Safety and Food Quality.* Mexican Government. Last access: December 2022 From: https://www.gob.mx/senasica/articulos/pequenos-productores-sosten-de-nuestra-agricultura

Schejtman, A. (1982) Economía campesina y agricultura empresarial. Tipología de productores del agro mexicano. CEPAL, Siglo XXI editores. México. Last access: december 2022, from: https://repositorio.cepal.org/handle/11362/25265

SIAP (2022). Information System of Agrifood and Fisheries (Sistema de Información Agroalimentaria y Pesquera SIAP). Ministry of Agriculture (Secretaría de Agricultura). Mexican Government. Last access: December 2022, From: https://www.gob.mx/siap

Toledo, V., Alarcón-Chaires, P., & Barón, L. (2002). *La modernización rural de México: un análisis socioecológico*. UNAM: México

UNCTAD (2015) *Commodities and Development Report 2015: Smallholder Farmers and Sustainable Commodity Development UNCTAD/SUC/2014/5,* United Nations Conference on Trade and Development, Geneva, Switzerland. Last access: December 2022, from: unctad.org/en/PublicationsLibrary/suc2014d5_en.pdf

Vargas Canales, J. M., Palacios Rangel, M. I., Camacho Vera, J. H., Aguilar Ávila, J., & Ocampo Ledesma, J. G. (2015). Factores de innovación en agricultura protegida en la región de Tulancingo, México. Revista mexicana de ciencias agrícolas, 6(4), 827-840. https://www.scielo.org.mx/scielo.php?script=sci_arttext&pid=S2007-09342015000400013

Yúnez, A., Cisneros, A. & Meza, P. (2013) Situando la agricultura familiar en México. Principales características y tipología. Serie Documentos de Trabajo N°149. Grupo de Trabajo: Desarrollo con Cohesión Territorial. Programa Cohesión Territorial para el Desarrollo. Rimisp, Santiago, Chile. Last access: december 2022, from: https://www.rimisp.org/documentos/situando-la-agricultura-familiar-en-mexico-principales-caracteristicas-y-tipologia/

Wiggins S, Kirsten J., & Llambi L. (2010) The future of small farms *World Development.* 38 1341–8. https://doi.org/10.1016/j.worlddev.2009.06.013

World Bank 2007 *World Development Report 2008: Agriculture for Development* (Washington, DC: The World Bank). Last access: December 2022, From: https://elibrary.worldbank.org/doi/abs/10.1596/978-0-8213-6807-7

Appendix 1: Mexican agricultural production and domestic supply of the 29 agricultural products analysed in this study in million tonnes, for the year 2019, compared with the total values reported by the FAO. Source of data: calculations by the authors using FAO (2022c) for agricultural production values (data of "crops primary" and "livestock primary"), and FAO (2022a) for domestic supply values. Note that FAO does not give values for amaranth and alfalfa. The details on the values with decimal points are shown in the Supplementary Information.

Table A1. Agricultural Production

Name of agricultural product according to each data source		Production [Million tons]
**Crops and livestock products (FAO, 2022c)**	**INEGI (2019)** (food group used in this study)	
Maize (corn)	white & yellow maize	27.23
Wheat	wheat	3.24
Rice	rice	0.25
Amaranth	amaranth	no data
Beans, dry	bean	0.88
Pumpkins, squash and gourds	squash	0.82
Onions and shallots, dry (excluding dehydrated)	onion	1.49
Chillies and peppers, green (Capsicum spp. and Pimenta spp.)	chilli pepper	2.72
Tomatoes	tomato	4.27
Avocados	avocado	2.30
Strawberries	strawberry	0.86
Lemons and limes	lemon	2.70
Mangoes, guavas and mangosteens	mango	2.40
Apples	apple	0.76
Oranges	orange	4.74
Bananas	banana	2.40
Grapes	grape	0.47
Sorghum	sorghum	4.35
Soya beans	soybean	0.23
Raw milk of cattle	milk	12.28
Meat of cattle with the bone, fresh or chilled	cattle meat	2.03

Meat of pig with the bone, fresh or chilled	pork	1.60
Meat of chickens, fresh or chilled	chicken meat	3.48
Hen eggs in shell, fresh	eggs	2.95
sugar cane	sugar cane	59.33
Coffee, green	coffee	0.17
Cocoa beans	cocoa	0.03
TOTAL for the 29 agricultural products included in this study:		143.97
Total "crops primary" and "livestock primary" reported by the FAO (2022c)		166.97
Share of the 29 food products included in this study in Mexico's total agricultural production		87%

Table A2. Domestic supply:

**Name of agricultural product according to each data source**		**[Million tons]**
**Food Balance Sheets (FAO, 2022a)**	**INEGI (2019) (food group used in this study)**	**Domestic supply** [Million tons]
Wheat and products	wheat	6.84
Rice and products	rice	1.41
Maize and products	white and yellow maize	47.13
Beans	bean	1.11
Tomatoes and products	tomato	2.56
Onions	onion	1.27
Vegetables, other	chilli pepper, squash	5.91
Lemons, Limes and products	lemon	1.73
Apples and products	apple	0.93
Oranges, Mandarines	orange	4.63
Bananas	banana	1.83
Grapes and products (excl wine)	grape	0.39
Fruits, other	avocado, strawberry, mango	6.11
Sugar cane	sugar cane	59.33

Coffee and products	coffee	0.08
Cocoa Beans and products	cocoa	0.09
Sorghum and products	sorghum	5.20
Soyabeans	soybean	4.78
Bovine Meat	cattle meat	1.90
Pigmeat	pork	2.34
Poultry Meat	chicken meat	4.65
Eggs	eggs	3.00
Milk - Excluding Butter	milk	13.49
TOTAL amount in tonnes included in this study		176.73
Total domestic supply reported by the FAO (2022a)		210.44
Share of total included in this study		84%

Appendix 2. Market and non-market production by each type of farm and each type of crop. Calculations by the authors using INEGI (2019a)

The following tables show the share (per farm and crop) of the production and the total production in tons that was sold ("market") and not sold ("no market") by the farms. The "no market" production includes the seeds that farms use for the following farming season, the food used for home consumption, and the feed used for livestock. The details of the share and production for seeds, food and feed, as well as the values with decimal points, are shown in the Supplementary Information.

Table A3. Total production of no market and market production produced by each type of farm and each type of crop. Source of data: calculations by the authors using INEGI (2019a)

Tons	Small farms		Medium farms		Large farms	
	Not market	market	Not market	market	Not market	market
Avocado	135	743,790	569	823,376	1,687	445,549
Alfalfa	863,822	1,694,021	171,608	1,324,157	484,387	1,070,391
Amaranth	8	567	69	1,020	223	3,466
Rice	868	107,858	183	39,057	23	9,443
Cocoa	46	3,229	1,192	5,407	1,749	12,644
Coffee	576	102,597	4,052	147,556	12,465	213,991
Squash	1,108	393,283	399	124,937	287	109,682
Sugar Cane	45,596	14,855,458	42,287	24,000,000	65,156	14,337,786
Onion	50	826,122	9	129,075	114	43,953
Chilli pepper	449	800,517	1,019	604,318	574	47,899
Strawberry	-	21,574	4	14,085	14	8,085
Bean	44,175	465,147	40,011	226,473	17,751	34,507
Tomato	907	893,758	1,265	121,576	162	57,949
Lemon	279	912,147	131	307,623	573	130,303
Yellow maize	521,165	3,500,598	247,125	694,573	197,026	240,494
White maize	1,231,565	10,724,702	1,152,651	8,453,322	1,261,406	2,987,188
Mango	325	473,256	2,584	253,098	1,152	184,598
Apple	157	222,431	5	147,663	223	22,376
Orange	1,370	1,617,578	3,261	1,232,379	3,148	642,371
Banana	438	1,499,405	555	140,201	1,296	285,591
Sorghum	193,156	2,199,371	56,589	869,511	13,056	339,386
Soybean	674	271,210	303	33,767	0	4,458
Wheat	16,881	3,328,673	2,338	367,088	280	19,831
Grape	47	283,532	15	20,745	-	-

Table A4. Share of no market and market production produced by each type of farm and each type of crop

	Small farms		Medium farms		Large farms	
	No market	market	No market	market	No market	market
Avocado	0.02%	99.98%	0.07%	99.93%	0.38%	99.62%
Alfalfa	33.77%	66.23%	11.47%	88.53%	31.15%	68.85%
Amaranth	1.33%	98.67%	6.33%	93.67%	6.04%	93.96%
Rice	0.80%	99.20%	0.47%	99.53%	0.24%	99.76%
Cocoa	1.39%	98.61%	18.06%	81.94%	12.15%	87.85%

Coffee	0.56%	99.44%	2.67%	97.33%	5.50%	94.50%
Squash	0.28%	99.72%	0.32%	99.68%	0.26%	99.74%
Sugar Cane	0.31%	99.69%	0.18%	99.82%	0.45%	99.55%
Onion	0.01%	99.99%	0.01%	99.99%	0.26%	99.74%
Chilli pepper	0.06%	99.94%	0.17%	99.83%	1.18%	98.82%
Strawberry	0.00%	100.00%	0.03%	99.97%	0.17%	99.83%
Bean	8.67%	91.33%	15.01%	84.99%	33.97%	66.03%
Tomato	0.10%	99.90%	1.03%	98.97%	0.28%	99.72%
Lemon	0.03%	99.97%	0.04%	99.96%	0.44%	99.56%
Yellow maize	12.96%	87.04%	26.24%	73.76%	45.03%	54.97%
White maize	10.30%	89.70%	12.00%	88.00%	29.69%	70.31%
Mango	0.07%	99.93%	1.01%	98.99%	0.62%	99.38%
Apple	0.07%	99.93%	0.00%	100.00%	0.99%	99.01%
Orange	0.08%	99.92%	0.26%	99.74%	0.49%	99.51%
Banana	0.03%	99.97%	0.39%	99.61%	0.45%	99.55%
Sorghum	8.07%	91.93%	6.11%	93.89%	3.70%	96.30%
Soybean	0.25%	99.75%	0.89%	99.11%	0.00%	100.00%
Wheat	0.50%	99.50%	0.63%	99.37%	1.39%	98.61%
Grape	0.02%	99.98%	0.07%	99.93%	0.00%	0.00%

Appendix 3. Imports and exports of agricultural products estimated for this study. Calculations by the authors using INEGI (2019a) and FAO (2022b).

The FAO gives data of exports and imports (FAO, 2022b) and production values (FAO, 2022c) of all primary products. The values of production (FAO, 2022c) are slightly different from the values reported by the INEGI (2019a) (values of this paper) due to differences in methodology and type of agricultural products used in each data source. For this reason, for this study we calculated a "ratio value" of exports and imports using FAO data (table A4). This ratio was used to calculate the exports and imports for the 29 food groups of this study. Table A5 shows the results of this calculation.

The FAO does not distinguish between white and yellow maize. So, yellow and white maize production and trade values were taken from SAGARPA (2017). The details on the values with decimal points are shown in the Supplementary Information.

Table A5. Estimation of exports and imports ratio value. Data source: Production (FAO, 2022c), imports and exports (FAO 2022b).

**Name of agricultural product according to each data source**		Production (FAO, 2022c)	Exports (FAO 2022b)	Imports (FAO 2022b)	Ratio of exports	Ratio of imports
**FAO (2022b,2022c)**	**INEGI (2019)** (food group used in this study)					
	White maize	24.56	1.499	1.066	0.06	0.04
Wheat	Wheat	3.24	0.74	4.80	0.23	1.48
Rice, paddy (rice milled eq)	Rice	0.16	0.01	0.97	0.06	5.95
	Amaranth	no data	no data	no data	-	-
beans, dry	Bean	0.88	0.04	0.12	0.05	0.14
sugar cane	Sugar cane	no data	no data	no data	-	-
Pumpkins, squash and gourds	Squash	0.68	0.54	0.00	0.79	-
Onions, dry	Onion	1.49	0.35	0.13	0.24	0.09
Chillies and peppers, green	Chili pepper	3.24	1.07	0.00	0.33	0.00
Tomatoes	Tomato	4.27	1.86	0.00	0.44	0.00
Avocados	Avocado	2.30	1.15	0.00	0.50	-
Strawberries	Strawberry	0.86	0.14	0.02	0.16	0.02
Lemons and limes	Lemon	2.70	0.77	0.00	0.28	0.00
Mangoes, mangosteens, guavas	Mango	2.40	0.41	0.00	0.17	0.00
Apples	Apple	0.76	0.00	0.25	0.00	0.33
Oranges	Orange	4.74	0.06	0.03	0.01	0.01
Bananas	Banana	2.40	0.57	0.00	0.24	0.00

Grapes	Grape	0.47	0.22	0.09	0.46	0.19
Cocoa, beans	Cocoa	0.03	0.00	0.05	0.00	1.64
Coffee, green	Coffee	0.17	0.10	0.04	0.59	0.24
	Yellow maize	3.55	0.035	12.95	0.01	3.65
Alfalfa meal and pellets	Alfalfa	0.0	0.0	0.0	0.0	0.0
Sorghum	Sorghum	4.35	0.00	0.74	0.00	0.17
Soybeans	Soybean	0.23	0.00	4.85	0.01	20.85
Milk, whole fresh cow	Milk	12.28	0.00	0.21	0.00	0.02
Meat, cattle	Cattle meat	2.03	0.10	0.00	0.05	0.00
Meat, pig	Pork	1.60	0.00	0.70	0.00	0.44
Meat, chicken	Chicken meat	3.48	0.00	0.97	-	0.28
Eggs, hen, in shell	Eggs	2.95	0.00	0.05	-	0.02

Table A6. Production, exports, imports, and domestic supply values. Data source: Calculations by the authors.

**[Million tons]**	Production	Exports	Imports	Domestic Supply
White maize	25.81	1.58	1.12	25.36
Wheat	3.74	0.85	5.53	8.42
Rice	0.16	0.01	0.94	1.08
Amaranth	0.01	0.00	0.00	0.01
Bean	0.83	0.04	0.12	0.90
Sugar cane	53.25	0.00	0.00	53.25
Squash	0.64	0.50	0.00	0.13
Onion	1.00	0.24	0.09	0.85
Chilli pepper	2.00	0.66	0.00	1.34
Tomato	2.86	1.24	0.00	1.62
Avocado	2.02	1.01	0.00	1.01
Strawberry	0.23	0.04	0.00	0.20
Lemon	1.35	0.38	0.00	0.97
Mango	0.92	0.16	0.00	0.76
Apple	0.48	0.00	0.16	0.64
Orange	3.50	0.04	0.02	3.48

Banana	1.93	0.46	0.00	1.47
Grape	0.32	0.15	0.06	0.23
Cocoa	0.02	0.00	0.04	0.06
Coffee	0.48	0.29	0.12	0.31
Yellow maize grain	5.40	0.05	19.70	25.05
Alfalfa	5.61	0.00	0.00	5.61
Sorghum	3.67	0.00	0.63	4.30
Soybean	0.31	0.00	6.48	6.78
Milk	12.49	0.00	0.21	12.70
Cattle meat	2.03	0.10	0.00	1.93
Pork	1.60	0.00	0.70	2.30
Chicken meat	3.48	0.00	0.97	4.45
Eggs	2.95	0.00	0.05	3.00
TOTAL	139.08	7.80	36.95	168.22

Appendix 4. Distribution of agricultural products production by type of farm in Mexico.

Values are given in million tons, the share of the production per type of farms indicated in parenthesis. Source: calculations by the authors. See section 3.1 for details.

Table A7. Distribution of agricultural products production by type of farm in Mexico.

Mt (%)	Small scale	Medium scale	Large scale	Protected agriculture	Total
White maize	4.0 (17%)	9.0 (37%)	11.2 (46%)	0.0 (0%)	24.2 (100%)
Other cereals	0.0 (1%)	0.3 (11%)	2.7 (88%)	0.0 (0%)	3.0 (100%)
Beans	0.0 (6%)	0.3 (32%)	0.5 (62%)	0.0 (0%)	0.8 (100%)
Vegetables	0.1 (3%)	0.6 (15%)	1.8 (46%)	1.4 (36%)	3.8 (100%)
Fruits	1.4 (16%)	2.3 (28%)	4.6 (53%)	0.2 (3%)	8.5 (100%)
Animal feed	2.3 (16%)	3.4 (23%)	9.2 (61%)	0.0 (0%)	14.9 (100%)
Animal products	3.2 (14%)	3.9 (18%)	15.3 (68%)	N.A.	22.4 (100%)
Stimulants	0.1 (48%)	0.1 (31%)	0.0 (20%)	0.0 (1%)	0.2 (100%)
Sugar cane	14.3 (27%)	24.0 (45%)	14.9 (28%)	0.0 (0%)	53.3 (100%)
TOTAL	25.6 (19%)	43.9 (33%)	60.1 (46%)	1.6 (2%)	131.3 (100%)

Figure1

Click here to access/download;Figure;figure 1.pdf

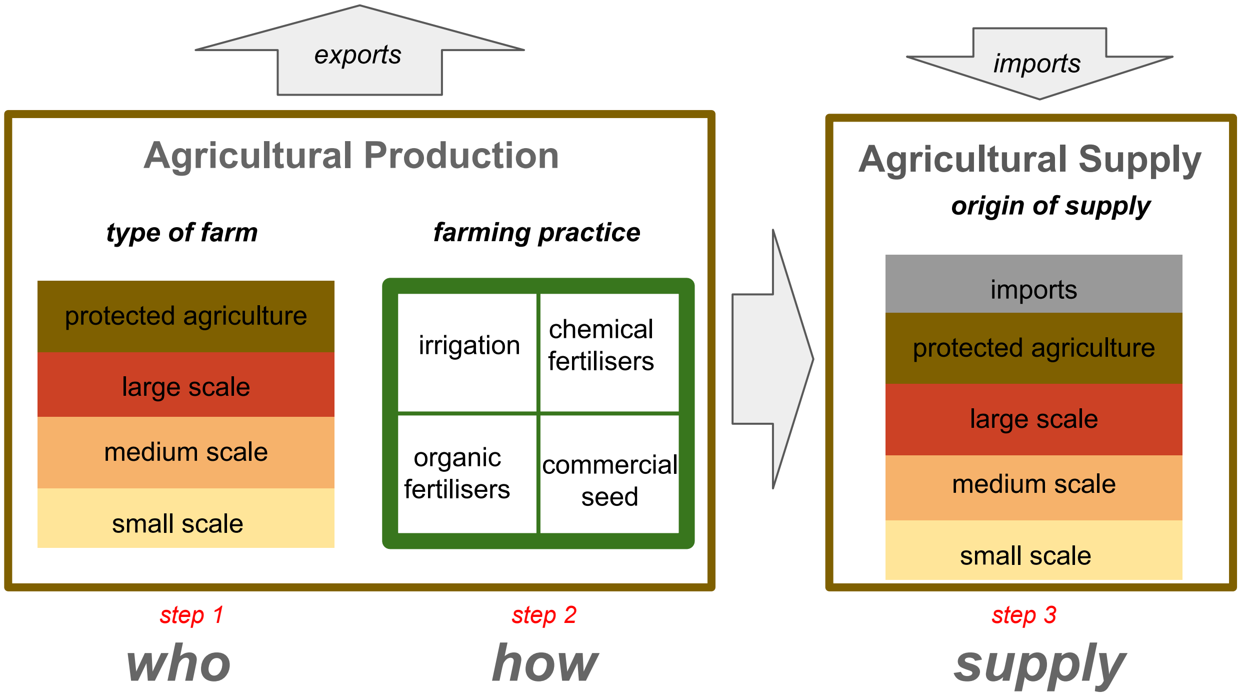

Click here to access/download
Supporting Information
Supplementary Information.xlsx

Title: How much of the Mexican agricultural supply is produced by small farms, and how?

Authors: María-José Ibarrola-Rivas [1, a]; Quetzalcóatl Orozco-Ramírez [2]; Louise Guibrunet [3]

Authors information:

[1] Physics Geography Department, Geography Institute, Autonomous National University of Mexico (UNAM), Mexico City, Mexico. ibarrola@geografia.unam.mx ORCID: 0000-0003-4501-0408

[2] Academic Unit campus Oaxaca, Geography Institute, Autonomous National University of Mexico (UNAM), Oaxaca, México. qorozco@geografia.unam.mx ORCID: 0000-0002-3085-7406

[3] Social Geography Department, Geography Institute, Autonomous National University of Mexico (UNAM), Mexico City, Mexico louiseg@geografia.unam.mx ORCID 0000-0002-1377-4852

Corresponding author: [a] Post Address: Institute of Geography UNAM. Circuito de la Investigación Científica S.N., Ciudad Universitaria, Coyoacán, C.P. 04510 Ciudad de México, CDMX

Acknowledgements The data used in this paper were processed and analyzed under project number LM-530 at the Microdata Laboratory of the National Institute of Statistics and Geography (INEGI) at their offices in Mexico City. The authors give special thanks to the staff of INEGI Microdata Laboratory (María de Lourdes Garrido Blancas, Lidia Hernández Hernández, Liliana Martínez Matias, Andrea López Ortiz, and Natalia Eugenia Volkow Fernández) and the experts of the agricultural census and survey at INEGI (Victor Dueñas de la Rosa, Karina Lara Fernández, Luis Fernando Esteves Cano, Ismael Saucedo Silva) for their unconditional support during our work at the Microdata Laboratory, even in times of pandemic.

Funding This project was partly funded by two PAPIIT (*Programa de Apoyo a Proyectos de Investigación e Innovación Tecnológica*) projects of the National Autonomous University of Mexico with projects numbers: IN300322 for Ibarrola-Rivas and IA300222 for Guibrunet. The funders had no role in study design, data collection and analysis, decision to publish, or preparation of the manuscript.

How much of the Mexican agricultural supply is produced by small farms, and how?

Abstract

The contribution of small farms to the global food supply is in debate due to lack of empirical evidence. In Mexico, small farms have been relatively important for national food supply due to an agrarian reform in the first half of the 20th century, but their role has been decreasing in the last decades. The aim of this study is to quantify how much small farms produce of the Mexican agricultural supply, and with which farming practices, using the 2019 National Agricultural Survey. The results show that small farms produce 19% of the national agricultural production with similar farming practices to those of medium and large farms. When considering imports and exports, small farms produce 15% of the national agricultural supply. [24] The production of small farms consists mainly of cash crops (e.g. sugar cane, fruits & vegetables, animal products, fodder crops) and, to a lesser extent, staple crops such as maize and beans. The fact that small farms produce one fifth of the national production after decades of governmental support towards large farms suggests that they have resilient production systems. The results of this study support that stronger efforts should be made to enhance the role of small farms in achieving Mexican food sovereignty. This will not only have benefits in terms of food supply but may also have a wide range of social and environmental benefits.

Keywords: Agricultural supply; small farms; Mexico; farming practices; peasants;

1. Introduction

Attention to the contribution of small farmers to global food supply and to agricultural sustainability is increasing (UNCTAD, 2015; Wiggins et al, 2010; World Bank, 2007; IFAD and UNEP, 2013). Small farms have been associated with beneficial agricultural practices such as seed saving (McGuire and Sperling, 2016), with an agricultural production that relies on little to no external inputs, and with generating livelihoods and reducing poverty (Wiggins et al, 2010, de

Janvry & Sadoulet, 2022). These characteristics of small farms imply that they contribute to the environmental sustainability of the global food system (IFAD and UNEP, 2013; UNCTAD, 2015) and to food sovereignty (Altieri et al., 2012).

Small farms represent 90% of all farms worldwide and provide direct or indirect livelihoods to up to 2.5 billion people (UNCTAD, 2015). Yet, their contribution to the global food supply remains contested. Many documents have claimed that small farms produce 70% to 80% of the global food supply (though this claim is often unsupported by empirical evidence) (Ricciardi et al, 2018). For instance, a recent report states that small farms "provide over 80 per cent of the food consumed in a large part of the developing world" and "the bulk of food in developing countries" (IFAD and UNEP, 2013, pp.6,10). The report cites two sources of evidence to support these claims, one of which is an unpublished speech to the World Agricultural Heritage Forum by its founder and president (also former FAO senior employee) (Koohafkan, 2011). The other is an opinion piece by the president of the International Fund for Agricultural Development (IFAD, 2011), where the same figure is stated without any references. This statement can therefore not be traced back to any evidence or empirical study that can support this claim.

In the face of this unsupported claim that small farms produce most of the global food supply, recent studies have used robust datasets and analytical methods to provide estimates of the share of small farms' production in food supply globally and in different regions. For Ricciardi et al., (2018), small farms (<2ha) produce approximately one third of the global food supply. A similar figure is found in a recent FAO report, which specifies that small farms (<2ha) produce 36% of the world's food (Lowder et al., 2019). Unsurprisingly, these estimates also vary regionally, and depending on the definition that is used to characterise small farms: for Herrero et al. (2017), very small farms (<2ha) contribute to about 30% of the supply of "most food commodities" in Sub-Saharan Africa, Southeast Asia, and South Asia. Samberg et al. (2016, p.1), on the other hand, argue that small farms (<5ha) in Latin America, sub-Saharan Africa, and South and East Asia "are

responsible for more than half of the food calories produced globally, as well as more than half of global production of several major food crops". A common conclusion of these studies is that further research is needed to quantify the role of small farms to the food supply of different regions.

To partially address this research gap, in this study, we quantify the contribution of small farms to Mexico's food supply. Mexico has a large diversity of farms in terms of size, socioeconomic, cultural, technological, and production characteristics ([5] FAO, 2018; de Ita, 2014; [4] Schejtman, 1982; Yunez et al., 2013). Mexican studies on rurality have traditionally distinguished two large categories of farms: peasants and business farms ([4] Schejtman, 1982; Toledo et al., 2002; de Ita, 2014). Peasants (or small farmers) are usually defined as those that farm less than 2 ha or 5 ha of cropland, and as having specific characteristics such as consuming part of their production, relying on family labour, using few or no external inputs (such as [14] inorganic fertilisers or pesticides), and having a low income ([4] Schejtman, 1982; Toledo et al., 2002, de Ita, 2014).

The reality of Mexican farms is more complex than this categorisation suggests: a large spectrum of farms exists beyond peasants and business farms, and criteria for categorizing farms vary according to different authors. Schetjman (1982) defined four categories of farms: peasants (relying on family labour), transition farmers (relying on both family and salaried labour, but mainly the second), agricultural entrepreneurs (only salaried labour), and livestock companies (only animal production). For peasant farms he distinguished four categories: infra subsistence (< 4 ha), subsistence (4 - 8 ha), stationary (8-12 ha), excedentary (> 12 ha). For agricultural entrepreneurs he defined three types: small (500 - 1250 labour days per year) medium (1250 - 2500 labour days per year) and large (> 2500 labour days). For livestock companies the types were: small (< 50 cattle heads or equivalent), medium (50 - 300 cattle heads or equivalent), and large (>300 cattle heads or equivalent). Toledo et al (2002) used a socioecological analysis to define an index composed of nine indicators to describe a farm, or any territorial unit in terms of

agriculture type. The typology moves along a scale that goes from peasant to agroindustrial agriculture. The nine criteria used in their analysis are: energy, scale, self-sufficiency, labour (family vs salaried), diversity of crops, labour productivity, energy productivity, type of knowledge used, and cosmovision. For Toledo et al. (2022), farms practising rainfed agriculture on less than 5 hectares are peasant and larger farms or with irrigation no matter the size are agroindustrial. For Yunez et al. (2013), a family farm is one that uses more than 50% of family labour. They also separate specialised family farms, when more than 50% of the income comes from farming, and pluriactivity family farms, when farming income is less than 50%. For de Ita (2014), the two criteria for farm classification are type of labour and size. Peasant farms only use family labour and the size is less than 20 ha. Entrepreneur farms use salary labour and are larger than 20 ha. As we can see, criteria can vary in all these typologies.

Diverse benefits of Mexican small farms have been documented in the literature. Firstly, they contribute to national food security (Galeana-Pizaña et al, 2021) and provide 60% of agricultural labour (FAO, 2018). Yet, quantifications of the contribution of small farms to the domestic supply are lacking. The Mexican government estimates that small farms (<5ha) produce 40% of the country's food supply (SENASICA, 2021), though they do not explain the methodology used to calculate this figure. Secondly, small farms contribute to agrobiodiversity, including genetic and food diversity, as well as associated cultural diversity (de Ita, 2014; Yunez et al., 2013; FAO 2018, Astier et al., 2021). Because they tend to maintain traditional agroecological practices, small farms are thought to be more resource efficient and hence more environmentally sustainable than larger farms ([6] Bustamante et al., 2017).

Mexico's agrarian reform following the Mexican revolution in 1910 distributed almost half of the country's land as communal land (ejidos) to small farmers (Sanchez, 2003). With this, land tenure was recognized to indigenous communities resulting in (1) peasant's food production as the basis of the national production, and (2) preserving traditional practices ([7] Sánchez, 2003), unlike

other Latin American countries which did not have extended agrarian reform and are characterised by unequal land distribution (Kay, 1999). However, this tradition of peasant farming and small farms has been displaced, to a certain extent, by the green revolution of the second half of the twentieth century (de Gortari Rabiela, 2020) and more recently by the impacts of the North American Free Trade Agreement (González, 2006). Since the 1990's, Mexican neoliberal reforms have focused on supporting large-scale and agroindustrial agriculture, thus displacing small farmers (de Ita, 2014). The federal government has recently started to revert this trend, with governmental programs supporting small farmers with technical and financial resources as a double strategy to reach national food sovereignty and to alleviate rural poverty (DOF, 2020; SADER, 2017). This strategy could be enhanced by robust and recent studies that analyze the characteristics of small farms, including their agricultural practices and the share of their production in the Mexican food supply to discuss their potential role, and changes needed, to reach Mexican food sovereignty.

The aim of this study is to quantify how much of the agricultural supply in Mexico is produced by small farms, and to analyse the farming practices used to produce it. To do this, we answer the following research questions: (1) How much of the agricultural food products are produced in Mexico by farms of different sizes? (2) What farming practices are used by farms of different sizes? (3) How much of the agricultural supply is produced by small farmers? With this, we discuss the role of small farmers for food sovereignty, and some implications of their use of different farming practices. The insights of this paper can be used to design agricultural programs to support small farmers as a means to reach national food sovereignty.

2. Material and methods

System description and analytical steps

[3] The system of this paper is composed of two elements (agricultural production and agricultural supply), which are analysed in three separate steps: type of farm, farming practices, and origin of supply (Figure 1).

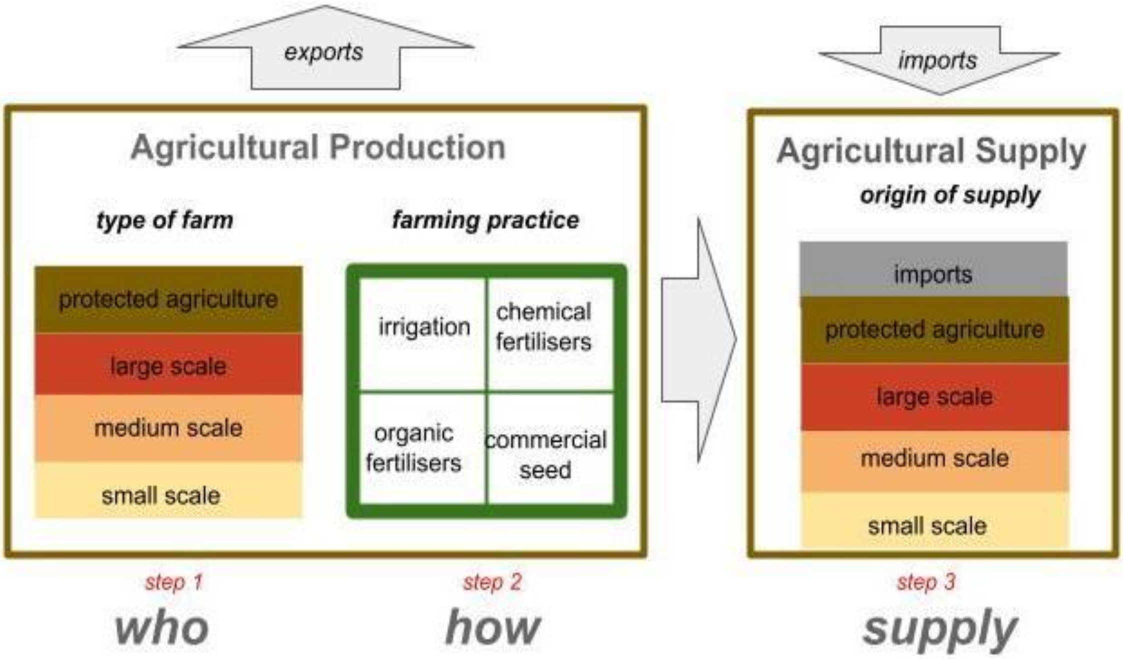

Figure 1. System description. Figure designed by the authors.

Table 1. Criteria for grouping the farms by type. The criteria are based on the sized-based strata defined by the survey (INEGI, 2019a).

Type of farm	*Criteria for each type of agricultural products*			
	**Crops**	**[12]Cattle products**	**pig products**	**poultry products**
**Small scale**	5 ha or less	≤ 26 animals	≤16 animals	≤ 500 animals
**Middle scale**	5 ha - 50 ha	27-120 animals	17-150 animals	500-20,000 animals
**Large scale**	> 50 ha	> 120 animals	>more than 150 animals	> 20,000 animals
**Protected agriculture**	Farms that use protection technologies (microtunnels or greenhouses) to produce crops.			

[3] NOTE: The survey groups cropland farms in six strata based on their cropland area, and livestock farms in four strata based on their number of animals. For crop farms, large-scale farms include strata 1 (50ha>x) and strata 2 (50ha> x <20ha); medium-scale farms include strata 3 (20ha> x <10ha) and strata 4 (10ha> x <5ha); and small-scale farms include strata 5 (5ha> x <2ha); and strata 6 (x<2). For cattle and pig farms, large-scale farms include strata 1 (120 cows >x & 150 pigs >x); mediums-scale farms include strata 2 (120 cows > x < 36 cows & 150 pigs> x <16 pigs); and small-scale farms include strata 3 (36 cows> x < 11 cows & 16 pigs> x < 3 pigs) and strata 4 (x<11 cows & x<2 pigs).

The Mexican agricultural production is the starting point of the analysis. It is defined as the total amount of agricultural products (crops and animal products) that Mexican farms produce (step 1). The criteria to categorise the type of farm are the area of cropland and the number of animals (Table 1), since these are the most common criteria in the literature (Chalate-Molina et al., 2010;

Herrero et al. 2017; [8] de Ita, 2014; Lowder et al., 2019; Ricciardi et al., 2018; Samberg et al. 2016; Schejtman, 1987). Protected agriculture is considered as a different category because, compared with non-protected agriculture, its production is mainly determined by investment, technology level, and knowledge of farmers about greenhouse operation (Moreno Reséndez et al., 2011; Vargas Canales et al., 2015), as well as because it can be practiced in areas unsuitable for open agriculture (Moreno Reséndez et al., 2011). [13]&[3] In addition, protected agriculture is more efficient in terms of resource use because of the use of intensive production systems with high yielding seeds and because the environmental factors can be controlled, thus reducing risks of losses and enabling crop production all year round (Jensen & Malter, 1995).

Then, we analysed the farming practices used by Mexican farms (step 2). Four indicators were used to define the farming practices: use of irrigation, use of [14] inorganic fertilizers, use of organic fertilizers, and use of commercial seeds.

Finally, we calculated how much of the national agricultural supply [3] (including imports and excluding exports) is produced by small farmers in Mexico (step 3). We use the concept of domestic supply defined by the FAO,[1] which includes all primary production available in the country as well as the losses in the food chain, to define the national agricultural supply (Figure 1).

Materials: Data source

The data source is the 2019 Mexican National Survey of Agriculture, produced periodically by the Mexican federal government (INEGI, 2019a). We use the microdata of the survey (a database

[1] The FAO definition of domestic supply includes the primary commodities produced in the country, minus exports, plus imports, plus changes in stock (FAO, 2001). For this study, the changes in stock are not considered. Note that the domestic supply includes food products such as agricultural products (crops and animal products) and processed foods (e.g. sugars, alcoholic beverages, and vegetable oils), and also non food products (e.g. crop's products for pharmaceutics & textiles), feed products, commodities for processed production, and losses in the food chain.

including the individual responses of each respondent). This dataset is not publicly available [1]&[3] to ensure confidentiality for the respondents. Access to process and analyse the microdata was granted by the National Institute of Statistics and Geography (INEGI) through its Laboratory of Microdata for research purposes.

The survey presents data for the 29 major agricultural products (Table 2). These 29 products, which are those considered in this study, account for 87% of the Mexican agricultural production and 84% of the Mexican domestic supply in 2019 (Appendix 1, data from FAO (2022a, 2022b, 2022c)).

Table 2. Agricultural products analysed in this study (categorised by food group). Source of data: authors' categorisation based on INEGI (2019a).

**Food group**	**Agricultural products**
white maize	white maize
other cereals	wheat, rice, amaranth
bean	bean
vegetables	squash, onion, chilli pepper, tomato
fruits	avocado, strawberry, lemon, mango, apple, orange, banana, grape
animal feed	yellow maize, alfalfa, sorghum, soybean
animal products	milk, [12]cattle meat, pork, chicken meat, eggs
stimulants	cocoa, coffee
sugar cane	sugar cane

[3] The survey was applied to 63,855 farms, which is a statistically representative sample for each of the food products and types of farms. Sampling weights were included in the original database (INEGI, 2019b). [1] In the survey, farms are categorized in six strata (crop farms) and four strata (livestock farms) based on their amount of cropland area and livestock per farm (INEGI, 2019a), see Table 1. The survey does not assign strata for poultry production; therefore the selection was based on expert knowledge (González-Cerón, 2022).

Methods: Data analysis

[1] *Crop farms*

[1] & [2] For farms that produce crops, each respondent reports the production and cultivated area per crop , [22] & [3] taking into account crops intended to be sold on the market, those kept as seeds for the next season, those used for feed, and those intended for home consumption. Farms with open agriculture (unlike those practising protected agriculture) report the share of their production that is dedicated to each use; this data is presented in Appendix 2. Using the sampling weights, these values are aggregated by crop and type of farm using the *"collapse (sum)"* function of STATA software. The results are shown in Table 3.

[1]& [3] *Livestock farms*

As part of the survey, each respondent reported the number of animals on the farm and its production purpose (e.g. [9] beef cows, laying hens, milking cows). We used this data, and its associated sampling weights to estimate the annual animal products production in each type of farm as follows.

[1] & [2]&[3] The total number of animals per type of farm was calculated with the *"collapse (sum)"* function of STATA software by aggregating each type of animal reported by type of farm using the sampling weights. Having the total animals in each type of farm, we estimated the share of animals in each farm (e.g. x% of national milking cows are in small-scale farms). This percentage was multiplied by the national annual production of beef, milk, pork, chicken meat, and eggs, respectively, reported by the Agriculture and Fishery Information System of Mexico in 2019 (SIAP, 2022). With this, we estimated the amount of each animal product produced by each type of farm. The results are shown in Table 3.

For beef production, we included [9] beef cows and half of the "dual" animals ([9] milk and beef producing cows). For milk production, we included the milking cows and half of the "dual"

animals. For pig production, we considered all pigs reported by each farm (any age). [3] The sample for poultry production farms is a deterministic sample which does not come with sampling weights, which means that the total national values cannot be quantified. To address this, we used the distribution of these farms among the small-, medium-, and large- scale farms to estimate the national distribution of these farms. The sample of the poultry production farms represent 90% of the national production of eggs and chicken meat (source: personal communication with the staff of the National Agricultural Surveys of INEGI). To estimate the poultry meat production, we considered growing chickens, and for egg production, we considered the hens reported by each farm.

[1] *Farming practices*

[3] Each respondent reported whether they used an irrigated or rainfed system, applied [14] inorganic fertilizers and/or organic fertilizers, and the type of seed used. [15] The organic fertilizers used by farmers are mainly manure of different livestock, compost, and other homemade fertilizers such as earthworm humus (INEGI, 2019a). For the type of seed, respondents could choose from four categories: (1) landrace or [10] farm saved seeds, (2) [11] improved (hybrid varieties or varieties that have been selected by a rigorous methods to assure quality, performance and yield if environmental conditions are optimal), (3) certified or registered, (4) genetically modified. We grouped the improved, certified or registered, and the genetically modified seeds as “commercial seeds”, and the landrace or farm saved seeds as “non commercial seeds”. These data were used to define four variables characterizing farming practices: (a) use of irrigation, (b) use of [14] inorganic fertilizers, (c) use of organic fertilizers, (d) use of commercial seeds. Farming practices of protected agriculture are not reported in the survey, so these farms were not considered in the analysis. This analysis also excluded animal products; but by including [12] fodder crops, we considered these products indirectly. [1] & [2] The answers of all respondents were aggregated per type of farm and type of farming practice,

using sampling weights, with the *"collapse (sum)"* function of STATA software. The results are shown in Figure 2.

[1] *Agricultural supply*

Finally, to calculate the agricultural supply, we quantified the exports and imports of the 29 agricultural products included in this study (Appendix 3). The exports were subtracted equally for all types of farms from the national production, and the imports were added as a new category making up the domestic supply (Figure 1, step 3). With this, we calculated how much of the agricultural supply is produced by small farms, farms of other sizes, or obtained from imports. See Appendix 3 for details.

3. Results

3.1 Agricultural production in Mexico by type of farm

Most of the food produced in Mexico (64%) comes from large farms (Table 3). Small farms produce 19%, medium-scale farms produce 33%, and protected agriculture produce 2% of the Mexican agricultural production. The share of the production of each type of agricultural product is also different among the four types of [12] farms (Appendix 4). Almost half of the white maize (the main food product in the Mexican diet) is produced by large-scale farms, and 17% is produced by small farms. Beans, also one of the main food products of the Mexican diet, are mainly produced by large-scale farms, followed by medium farms, while small farms produce 6% of the national production. Vegetables are mainly produced by large-scale farms, followed by protected agriculture farms (the latter producing almost exclusively vegetables). Animal products and [12] fodder crops are also mostly produced by large-scale farms (68% and 62%), followed by medium-scale farms (18% and 27%), and by small-scale farms (14% and 16%). Even though small-scale farms produce a relatively small share of the national production, they account for the largest share of farms (57% of crop farms, 78% of [12] cattle farms, 89% of pig

farms, and 96% of poultry farms) (Table 3). This difference in the number of farms of different sizes and their total production can be explained by the fact that the average production per farm is much higher in large scale farms, followed by medium- and small- scale farms (see 4th row in Table 3). Large-scale farms' average production is ten times higher than small-scale farms for crops, 70 times higher for [12] cattle farms, and 2000 times higher for pig farms. However, the average crop yield is similar among the three types of farms (Table 3). Protected agriculture, on the other hand, boasts yields ten times higher than all other farms - this can be explained by the fact that it produces almost exclusively vegetables, which yields are higher than for other types of crops (such as cereals).

Table 3. Distribution of farms, production, and cropland among the type of farms, and productivity indicators. Source: calculations by the authors using INEGI (2019a). [23] See Appendix 4 for data by food item and Supplementary Information for details [1]

		Small scale		Medium scale		Large scale		Protected agriculture		Total	
Number of Farms [Million farms]	Crops	1.97	*57%*	1.05	*30%*	0.43	*12%*	0.02	1%	3.46	*100%*
	[12]Cattle	1.09	*78%*	0.27	*19%*	0.04	*3%*	N.A.	N.A.	1.40	*100%*
	Pig	0.34	*17%*	0.04	*2%*	1.60	*81%*	N.A.	N.A.	1.98	*100%*
	Poultry	N.A.	*96%*	N.A.	*0%*	N.A.	*4%*	N.A.	N.A.	N.A.	*100%*
Production [Million tonnes]	Crops	23.27	*20%*	41.77	*36%*	48.86	*42%*	2.63	*2%*	116.53	*100%*
	[12]Cattle	3.09	*21%*	3.84	*26%*	7.59	*52%*	N.A.	N.A.	14.52	*100%*
	Pig	0.13	*8%*	0.13	*8%*	1.34	*84%*	N.A.	N.A.	1.60	*100%*
	Poultry	0.00	*0.0%*	0.00	*0.1%*	6.42	*99.9%*	N.A.	N.A.	6.43	*100%*
Cropland area [Mha]	Crops	3.27	*21%*	5.15	*33%*	7.24	*46%*	0.05	*0.3%*	15.71	*100%*
**Average farm Production [ton / farm]**	**Crops**	**11.83**		**39.89**		**114.61**		**145.69**		**33.69**	
	**[12]Cattle**	**2.83**		**14.43**		**199.59**		**N.A.**		**10.38**	
	**Pig**	**0.39**		**3.36**		**0.84**		**N.A.**		**0.81**	
	**Poultry**	**N.A.**		**N.A.**		**N.A.**		**N.A.**		**N.A.**	
**Average yield [ton/ha]**	**Crops**	**7.12**		**8.11**		**6.75**		**50.86**		**7.42**	
	**[12]Cattle**	**N.A.**		**N.A.**		**N.A.**		**N.A.**		**N.A.**	
	**Pig**	**N.A.**		**N.A.**		**N.A.**		**N.A.**		**N.A.**	
	**Poultry**	**N.A.**		**N.A.**		**N.A.**		**N.A.**		**N.A.**	

[22] The production values presented in Table 3 include products that are sold by farmers as well as those intended for other uses (animal feed, seeds and home consumption). Only 6%, 4% and 9 % of the production is not sold by farmers, for small-, medium and large-scale farms respectively (Appendix 4). This percentage diverges among food crops. For instance, less than

90% of maize, beans, and alfalfa are sold - across all farm types. This means that the tree type of farms use a similar share of their production of these crops for food, feed and seeds.

3.2 Farming practices used in the production

Farming practices are similar across the three types of farms (Figure 2). Over 90% of farms of any size use [14] inorganic fertilizers. Less than a quarter of farms (of any size) use organic fertilizers. This means that most crops are produced with [14] inorganic fertilizers and without organic fertilizers across all farm sizes. The use of irrigation systems shows a different pattern. It is mostly used by large-scale farms (66%) but is also commonly used by small- and medium-scale farms (53% and 47% respectively).

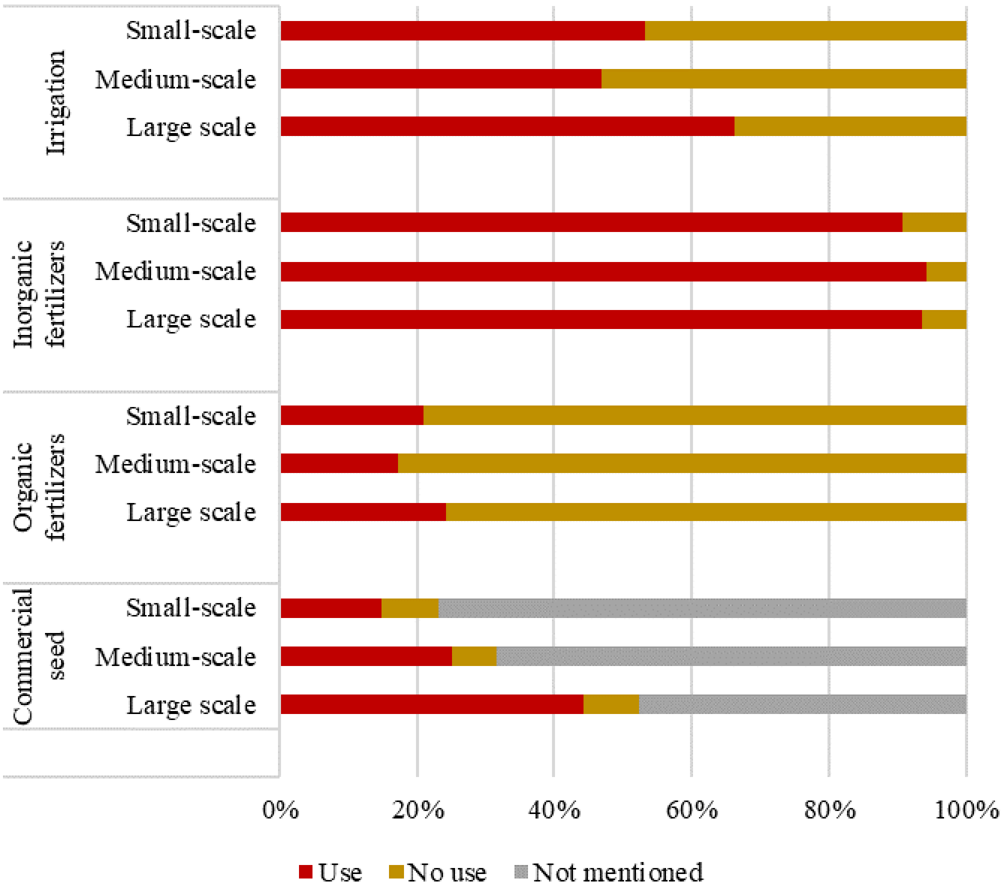

Figure 2. Farming practices used in the production of crops per type of farm. Source of data: calculations by the authors using the National Agricultural Survey (INEGI, 2019a). [23] The data plotted in this graph can be found in the Supplementary Information.

Most farms failed to report the type of seeds they use (see grey values in Figure 2). Only 23%, 32% and 52% of the small-, medium, and large-scale farms, respectively, reported the type of seed used. Therefore, more data is needed to discuss the distribution of the type of seeds among small-, medium-, and large scale farms.

3.3 How much of the agricultural supply is produced by small farms?

7.8 million tons out of the 139 million tons of the agricultural products produced in Mexico are exported, which accounts for 6% of the national production (Appendix 3, Table A6). These are mainly vegetables (2.6 million tonnes) and fruits (2.2 million tonnes). The imports of the agricultural products considered in this study represent 36.95 million tons per year and are mainly animal feed (26.8 million tons: mainly yellow maize) and other cereals for food (mainly wheat which amounts to 5.5 million tons). By adding the imports and subtracting the exports from the national agricultural production, we obtain the national supply of agricultural products, which amounts to 168 million tons in 2019. 22% of the supply originates from imports, 15% is produced by Mexican small-scale farms, 26% by Mexican medium-scale farms, 36% by large-scale farms, and only 1% by protected agriculture (Figure 3, Table 4, Appendix 3).

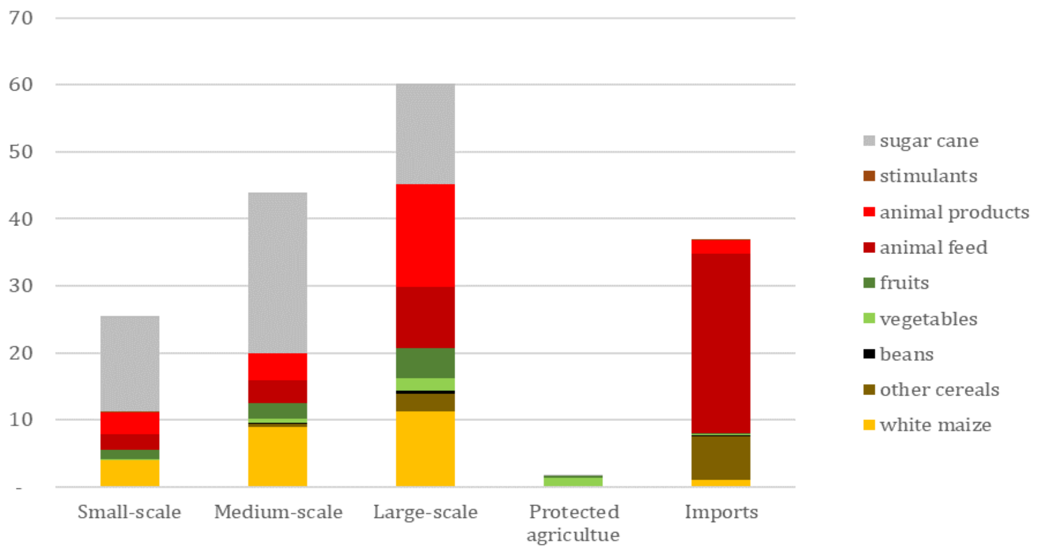

Figure 3. Source of the Mexican domestic supply in million tons, by food group. Source of data: calculations by the authors using the National Agricultural Survey (INEGI, 2019a) and FAO (2022b). [23] The data plotted in this graph can be found in Table A5, Appendix 4, and in the Supplementary Information.

Table 4. Source of national agricultural supply for each food product million tons and share of the total supply by food product (in italics). Source of data: calculations by the authors using the National Agricultural Survey (INEGI, 2019a) and [16] FAO (2022a). [23] The details on the values with decimal points are shown in the Supplementary Information.

Food group	Agricultural Product	Small scale	Medium scale	Large scale	Protected agriculture	Imports	Total
white maize	white maize grain	3.99	9.02	11.23	0.00	1.12	25.36
		16%	*36%*	*44%*	*0%*	*4%*	*100%*
other cereals	wheat	0.02	0.29	2.59	-	5.53	8.42
		0.2%	*3%*	*31%*	*0%*	*66%*	*100%*
	rice	0.01	0.04	0.10	-	0.94	1.08
		1%	*3%*	*10%*	*0%*	*86%*	*100%*[23]
	amaranth	0.004	0.001	0.001	0.000	-	0.01
		68%	*20%*	*11%*	*1%*	*0%*	*100%*[23]
	**TOTAL**	**0.03**	**0.32**	**2.69**	**0.00**	**6.47**	**9.51**
		0%	***4%***	***28%***	***0%***	***68%***	***100%***[23]
bean	bean	0.05	0.25	0.48	0.00	0.12	0.90
		5%	*28%*	*54%*	*0%*	*13%*	*100%*
vegetables	squash	0.02	0.03	0.08	0.00	-	0.13
		17%	*20%*	*62%*	*1%*	*0%*	*100%*
	onion	0.03	0.10	0.63	0.00	0.09	0.85
		4%	*12%*	*74%*	*0%*	*11%*	*100%*
	chilli pepper	0.03	0.41	0.54	0.36	0.00	1.34
		3%	*30%*	*40%*	*27%*	*0%*	*100%*[23]
	tomato	0.03	0.07	0.51	1.01	0.00	1.62
		2%	*4%*	*31%*	*63%*	*0%*	*100%*[23]
	**TOTAL**	**0.12**	**0.60**	**1.75**	**1.37**	**0.09**	**3.94**
		3%	***15%***	***45%***	***35%***	***2%***	***100%***
fruits	avocado	0.22	0.41	0.37	-	-	1.01
		22%	*41%*	*37%*	*0%*	*0%*	*100%*
	strawberry	0.01	0.01	0.02	0.16	0.00	0.20
		4%	*6%*	*9%*	*79%*	*2%*	*100%*[23]
	lemon	0.09	0.22	0.65	0.00	0.00	0.97
		10%	*23%*	*67%*	*0%*	*0%*	*100%*
	mango	0.15	0.21	0.39	0.00	0.00	0.76
		20%	*28%*	*52%*	*0%*	*0%*	*100%*
	apple	0.02	0.15	0.22	0.09	0.16	0.64
		3%	*23%*	*35%*	*14%*	*25%*	*100%*[23]
	orange	0.64	1.22	1.60	0.00	0.02	3.48
		18%	*35%*	*46%*	*0%*	*1%*	*100%*
	banana	0.22	0.11	1.14	0.00	0.00	1.47
		15%	*7%*	*78%*	*0%*	*0%*	*100%*
	grape	0.01	0.01	0.15	0.00	0.06	0.23
		3%	*5%*	*65%*	*1%*	*26%*	*100%*
	**TOTAL**	**1.36**	**2.34**	**4.55**	**0.25**	**0.25**	**8.75**
		15%	***27%***	***52%***	***3%***	***3%***	***100%***[23]

Table 4. ***continued.***

Food group	Agricultural Product	Small scale	Medium scale	Large scale	Protected agriculture	Imports	Total
animal feed	yellow maize grain	0.43	0.93	3.98	-	19.70	25.05
		2%	*4%*	*16%*	*0%*	*78%*	*100%* [23]
	alfalfa	1.55	1.50	2.56	0.00	-	5.61
		28%	*27%*	*45%*	*0%*	*0%*	*100%*[23]
	sorghum grain	0.35	0.93	2.39	-	0.63	4.30
		8%	*21%*	*56%*	*0%*	*15%*	*100%*[23]
	soybeans	0.00	0.03	0.27	0.00	6.48	6.78
		0%	*1%*	*4%*	*0%*	*95%*	*100%*[23]
	**TOTAL**	**2.34**	**3.39**	**9.20**	**0.00**	**26.80**	**41.74**
		**6%**	**8%**	**22%**	**0%**	**64%**	***100%***
animal products	milk	2.57	3.28	6.64	-	0.21	12.70
		20%	*26%*	*52%*	*0%*	*2%*	*100%*
	beef	0.50	0.53	0.90	-	0.00	1.93
		26%	27%	47%	0%	0%	*100%*
	pork	0.13	0.13	1.34	-	0.70	2.30
		6%	*5%*	*58%*	*0%*	*31%*	*100%* [23]
	chicken meat	0.00	0.00	3.47	-	0.97	4.45
		0%	*0%*	*78%*	*0%*	*22%*	*100%*
	eggs	0.00	0.00	2.95	-	0.05	3.00
		0%	*0%*	*98%*	*0%*	*2%*	*100%*
	**TOTAL**	**3.20**	**3.94**	**15.30**	**-**	**1.94**	**24.38**
		13%	***16%***	***63%***	***0%***	***8%***	***100%***
stimulants	cocoa	0.01	0.01	0.00	-	0.04	0.06
		23%	*10%*	*5%*	*0%*	*62%*	*100%*
	coffee	0.09	0.06	0.04	0.00	0.12	0.31
		29%	*20%*	*13%*	*1%*	*37%*	*100%*[23]
	**TOTAL**	**0.11**	**0.07**	**0.05**	**0.00**	**0.16**	**0.38**
		28%	***18%***	***12%***	***1%***	***41%***	***100%***[23]
sugar cane		14.35	24.00	14.90	0.00	-	53.25
		27%	*45%*	*28%*	*0%*	*0%*	*100%*
**Total agricultural supply**		**25.56**	**43.94**	**60.15**	**1.63**	**36.95**	**168.22**
		15%	***26%***	***36%***	***1%***	***22%***	***100%***

Different food products are sourced in different ways (Table 4). For instance, wheat, rice, animal feed, and cocoa are the food categories where imports represent more than half of the domestic supply. For these categories, the difference between the production of small-, medium-, and large-scale farms appears to be relatively less important when imports are considered. Only 0.2%, 1%, and 6% of the domestic supply of wheat, rice, and animal feed, respectively, is produced by small farmers.

The food products that are most largely produced by small farms (at least 20% of the domestic supply), in comparison with the other farms or imports, are amaranth (68%), avocado (22%), mango (20%), alfalfa (28%), milk (20%), beef (26%), sugar cane (27%), cocoa (22%), and coffee (29%). Al these products are considered “cash crops” or “commercial crops”, animal products or animal feed. None of them are staple foods. Also, production by small-scale farms never exceeds 30% of the national supply, apart from amaranth, but its production accounts to only 5.4 thousand tonnes in 2019, which is only 0.003 % of the total agricultural supply.

4. Discussion and conclusion

Mexican small farms, characterised in our study as those that have less than 5 hectares of [17] cropland, 16 pigs or less, 26 cows or less, or 500 chickens or less, produce 19% of Mexico’s agricultural production, and 15% of Mexico’s agricultural supply. The reduction in the percentage from production to supply is explained by the large quantities of food imports. These values differ from the widely reported statement that small farmers produce most of the food globally, and the Mexican government’s statement that small farmers produce 40% of the Mexican food (SENASICA, 2021). Recent studies (Ricciardi 2018 et al.; Samberg et al., 2016) estimating the global production of small farms have already shown a smaller estimate than the unsupported figure of 70%-80%. These authors estimate that small farms (under 5 hectares) produce 44%-48% (Ricciardi et al., 2018) and 55% (Samberg et al., 2016) of global food supply. Still, their results are much higher than the estimates of this study. This can partly be explained by the fact that small farms are more common to Africa and Asia than to Latin America (Samberg et al., 2016). This can also illustrate how Mexican small farms and the peasant farming traditions which had been preserved thanks to the agrarian reform of the early 20th century are losing importance as a result of the wave of neoliberal reforms of the last three decades (de Ita, 2014).

Similarly to Ricciardi et al (2018), our results show that farms of different sizes tend to produce different food products. Ricciardi et al. show that small farms produce mainly staple food crops (cereals, roots and tubers, pulses) and fruits, and large scale farms produce oil crops, vegetables, and other crops. However, we found a different pattern for the Mexican case. The main staple food crops for Mexican diets (white maize and beans) are mainly produced by large-scale farms. In contrast, the agricultural products that small farms most produce are “commercial” or “cash crops”: coffee and cocoa, sugar cane, followed by animal products, fruits, animal feed, and white maize; with only 16% of white maize being produced by small-scale farms (Table 4).

Further, farming practices do not differ widely according to the farm size (Figure 2). The farming practices of small-, medium-, and large- scale farms follow a similar pattern: most of the food is produced using [14] inorganic fertilizers, around half of farms of any size use irrigation, and a low share of the production relies on organic fertilizers. However, these findings must be corroborated and expanded through further research. In this analysis, we only considered a dichotomous characterisation of farming practices (whether farms use a certain agricultural input or not). But differences may exist in the amount or degree of use among the farms. For instance, small farms may use a smaller amount of fertilizer and irrigation than large scale farms. These differences must be quantified in a further study. In addition, the use of non-commercial seed has several social, cultural and agrobiodiversity implications. Efforts have been made to preserve diversity in Mexican seeds, particularly for native maize varieties which are preserved mostly by small farmers (Ortega, 2021). Unfortunately, as most farms fail to report the type of seed they use, our study does not allow us to [18] draw conclusions regarding the prevalence of the use of commercial seeds in Mexican farms. Further research is hence needed to identify the type of seeds used in Mexican agricultural production to enable discussions on the related agrobiodiversity, cultural and political implications of the use of different seeds.

Thus, our results show that Mexican small farms are not the main contributors to the Mexican agricultural supply, and their farming practices may have similar environmental implications than medium- and large- scale farms. However, these findings do not diminish the relevance of small farms for Mexico's food sovereignty and employment. Small farms represent the majority of farms in Mexico, a fifth of the cropland area, and the main provider of rural livelihoods. Small farms use marginal lands which otherwise would not be used by large-scale farms. In addition, they are a space for the generation of traditional knowledge and cultural heritage, which is a key tool for the resilience and adaptability of the national food system (Altieri & Nicholls, 2017; Altieri, 2004). Recently, some efforts have been made by the Mexican government to support small farmers (DOF, 2020), however they have not been enough. Since the 1990's, the governmental agricultural programs for small farmers have focused on increasing family income, rather than on enhancing production capacity (Yunez et al., 2013). The fact that small farms persist with little governmental support suggests that their production system is resilient due to their social land tenure ([19] de Ita, 2014), to their culture and cosmovision (Bartra 2020; [20] Leff 2020; Toledo 2002), and to their economic strategies (Bartra 2020). According to some authors (de Janvry & Sadoulet, 2022; Wiggins et al. 2010) small farms have the potential to increase their food productivity increased while reducing poverty.

To increase the relevance of small farms in Mexican food sovereignty, stronger efforts are needed to increase their role in the production of the main staple and nutritious food for the Mexican population, which will not only have benefits in terms of enhancing food sovereignty, but also may have a wide range of social and environmental benefits. This can be the focus of further agricultural programs promoting the production of nutritious staple food such as beans and white maize.

Conflict of Interests: The authors declared that they have no conflict of interest.

References

Altieri, M. A. (2004). Linking ecologists and traditional farmers in the search for sustainable agriculture. Frontiers in Ecology and the Environment, 2(1), 35-42. https://doi.org/10.1890/1540-9295(2004)002[0035:LEATFI]2.0.CO;2

Altieri, M. A., Funes-Monzote, F. R., & Petersen, P. (2012). Agroecologically efficient agricultural systems for smallholder farmers: contributions to food sovereignty. Agronomy for sustainable development, 32(1), 1-13. https://doi.org/10.1007/s13593-011-0065-6

Altieri, M. A., & Nicholls, C. I. (2017). The adaptation and mitigation potential of traditional agriculture in a changing climate. Climatic Change, 140(1), 33-45. https://doi.org/10.1007/s10584-013-0909-y

Astier, M., Perales Rivera, H., Orozco Ramírez, Q., Aragón Cuevas, F., Bye, R., Linares, E. y Mera Ovando, L. M. (2021). Conservación de la agrobiodiversidad en México: propuestas y experiencias en el campo. Comisión Nacional para el Conocimiento y Uso de la Biodiversidad (conabio) / Comisión Nacional de Áreas Naturales Protegidas (conanp). Ciudad de México. 212 pp. https://www.researchgate.net/publication/353851339_Conservacion_de_la_agrobiodiversidad_en_Mexico_Propuestas_y_experiencias_en_el_campo

Bartra, A. (2020) Repensar lo rústico, aportes a una teoría del campesinado contemporáneo. En: Boltvinik, J y Mann, S A. (Coords). Pobreza y persistencia campesina en el siglo XXI. pp 113-133. CROP, Editorial: Siglo Veintiuno Editores: México.

Bustamante, T., Carrera, B., & Schwentesius Rindermann, R. (2017). Sostenibilidad de pequeños productores en los Tianguis Orgánico de Tlaxcala, Puebla y Oaxaca, Veracruz. Estudios Regionales en Economía, Población y Región, 37(1), 1-33. https://doi.org/10.20983/epd.2017.37.1

Chalate-Molina, H., Gallardo-López, F., Pérez-Hernández, P., Paul Lang-Ovalle, F., Ortega-Jiménez, E., & Vilaboa Arroniz, J. (2010). Características del sistema de producción bovinos de doble propósito en el estado de Morelos, México. Zootecnia tropical, 28(3), 329-339. http://ve.scielo.org/scielo.php?script=sci_arttext&pid=S0798-72692010000300004

DOF, (2020) PROGRAMA Sectorial de Agricultura y Desarrollo Rural 2020-2024 (Sectoral Program for agriculture and rural development 2020-2024, Mexico). Federation. Diario Oficial de la Federación (Official Journal of the Mexican Federation). Last access: December 2022, from: https://dof.gob.mx/nota_detalle.php?codigo=5595549&fecha=25/06/2020

FAO, (2018) El México rural del siglo XXI. FAO. México. Last access: December 2022, from: https://www.fao.org/3/i9548es/I9548ES.pdf

FAO (2001) Concepts and definitions used In Food Balance Sheets. Food Balance Sheets, a Handbook. Food and Agricultural Organization of the United Nations, Rome 2001. Last access: January 2023, from https://www.fao.org/3/x9892e/X9892e02.htm

FAO, (2022a) Food Balance Sheets of the FAO. FAOSTAT. Last access: December 2022, from: https://www.fao.org/faostat/en/#data/FBS

FAO, (2022b) Detail Trade Matrix of the FAO. FAOSTAT. Last access: December 2022, from: https://www.fao.org/faostat/en/#data/TM

FAO, (2022c) Crops and livestock products. FAOSTAT. Last access: December 2022, from: https://www.fao.org/faostat/en/#data/QCL

Galeana-Pizaña, J. M., Couturier, S., Figueroa, D., & Jiménez, A. D. (2021). Is rural food security primarily associated with smallholder agriculture or with commercial agriculture?: An approach to the case of Mexico using structural equation modelling. Agricultural Systems, 190, 103091. https://doi.org/10.1016/j.agsy.2021.103091

Gonzalez, C. G. (2006). Markets, monocultures, and malnutrition: agricultural trade policy through an environmental justice lens. Michigan State Journal of International Law, 14, 345.

González Cerón, Fernando (2022) Personal communication with Dr. Fernando González Cerón, expert on Mexican poultry production. https://www.researchgate.net/profile/Fernando-Gonzalez-Ceron

de Gortari Rabiela, R. (2020). De la revolución verde a la agricultura sustentable en México. Nueva Antropología, 33(92), 66–86. Recuperado a partir de https://revistas.inah.gob.mx/index.php/nuevaantropologia/article/view/15995

Herrero, M., Thornton, P. K., Power, B., Bogard, J. R., Remans, R., Fritz, S., ... & Havlík, P. (2017) Farming and the geography of nutrient production for human use: a transdisciplinary analysis. The Lancet Planetary Health, 1(1), e33-e42. https://doi.org/10.1016/S2542-5196(17)30007-4

IFAD. 2011. Viewpoint: Smallholders can feed the world. Rome: International Fund for Agricultural Development. Last access: january 2023, From: https://www.ifad.org/documents/38714170/40706188/Smallholders+can+feed+the+world_e.pdf/460ca6c2-7621-40d8-9f79-a56f6f8fa75e

IFAD & UNEP, (2013) “Smallholders, food security, and the environment”. Report by the International Fund for Agricultural Development (IFAD) and the United Nations Environment Program (UNEP). Last access: december 2023 from: https://www.ifad.org/documents/38714170/39135645/smallholders_report.pdf/133e8903-0204-4e7d-a780-bca847933f2e

INEGI (2019a) Encuesta Nacional Agropecuaria 2019. Instituto Nacional de Estadística y Geografía (INEGI). Last access: June 2023 from: https://www.inegi.org.mx/programas/ena/2019/

[3] INEGI (2019b) Metodología Encuesta Nacional Agropecuaria 2019. Instituto Nacional de Estadística y Geografía (INEGI). Las access: June 2023 from: https://www.inegi.org.mx/app/biblioteca/ficha.html?upc=702825197315

De Ita, A. (2014). México: Economía campesina y agricultura empresarial, veinte años después. REVISTA ALASRU, 53. Last access: december 2022, from : https://www.ceccam.org/sites/default/files/Revista%20ALASRU.pdf#page=53

de Janvry, A., & Sadoulet, E. (2022). Agriculture for Development: Analytics and Action. Annual Review of Resource Economics, 14. 1-16. https://doi.org/10.1146/annurev-resource-090921-045011

[13] Jensen, M. H., & Malter, A. J. (1995). *Protected agriculture: a global review.* Washington D. C. World Bank. p.p. 157

Kay, C. (1999). Mirando hacia atrás: el tiempo de las reformas agrarias. Revista Envío, (208). https://www.envio.org.ni/articulo/952

Koohafkan, P. (2011) Globally important agricultural heritage systems. Presentation at the International Forum on Globally Important Agricultural Heritage Systems (GIAHS), Beijing, 9-12 June 2011

[20] Leff, E. (2020) De la persistencia del campesinado en el capitalismo al ambientalismo de los pueblos indígenas y la sostenibilidad de la vida. En: Boltvinik, J y Mann, S A. (Coords). Pobreza y persistencia campesina en el siglo XXI. pp 253-271. CROP, Siglo Veintiuno Editores: México.

Lowder, S.K., Sánchez, M.V. & Bertini, R. (2019) Farms, family farms, farmland distribution and farm labour: What do we know today? FAO Agricultural Development Economics Working Paper 19-08. Rome, FAO. Last access: December 2022, from: https://www.fao.org/agrifood-economics/publications/detail/en/c/1252236/

[21] ~~Lowder, S. K., Sánchez, M. V., & Bertini, R. (2021) Which farms feed the world and has farmland become more concentrated? World Development. 142. 105455, 1-15. https://doi.org/10.1016/j.worlddev.2021.105455~~

McGuire, S., & Sperling, L. (2016). Seed systems smallholder farmers use. Food Security, 8(1), 179-195. https://doi.org/10.1007/s12571-015-0528-8

Moreno Reséndez, A., Aguilar Durón, J., & Luévano González, A. (2011). Características de la agricultura protegida y su entorno en México. Revista Mexicana de agronegocios, 29(1345-2016-104296), 763-774. https://doi.org/10.22004/ag.econ.114479

Ortega, R. (2021). Estudios de diversidad, conservación in situ y mejoramiento de maíces nativos en México. Universidad Autónoma Chapingo, Texcoco, México.

Ricciardi, V., Ramankutty, N., Mehrabi, Z., Jarvis, L., & Chookolingo, B. (2018). How much of the world's food do smallholders produce?. Global food security, 17, 64-72. https://doi.org/10.1016/j.gfs.2018.05.002

SADER (2017) “Programa Apoyos a Pequeños Productores, campo joven y con raíces” Blog of the Ministry of Agriculture and Rural Development (Secretaría de Agricultura y Desarrollo Rural SADER). Last access: January 2023, from: https://www.gob.mx/agricultura/es/articulos/programa-apoyos-a-pequenos-productores-campo-joven-y-con-raices#:~:text=El%20programa%20de%20Apoyos%20a,peque%C3%B1os%20productores%20con%20servicios%20de

SAGARPA, (2017). Planeación Agrícola Nacional 2017-2030 (National Agricultural Plan 2017-2030). Ministry of Agriculture, Rural Development, Fishery and Food (Secretaría de Agricultura, Ganadería, Desarrollo Rural, Pesca y Alimentación). (SAGARPA)Last Access: January 2023, From: https://www.gob.mx/cms/uploads/attachment/file/255627/Planeaci_n_Agr_cola_Nacional_2017-2030-_parte_uno.pdf

Samberg, L. H., Gerber, J. S., Ramankutty, N., Herrero, M., & West, P. C. (2016). Subnational distribution of average farm size and smallholder contributions to global food production. Environmental Research Letters, 11(12), 124010. http://doi.org/10.1088/1748-9326/11/12/124010

Sánchez, J. C. M. (2003). Reforma agraria: del latifundio al neoliberalismo. Ed. Plaza y Valdés. p.p. 238. ISBN: 970-722-180-1

SENASICA (2021) Blog de: Servicio Nacional de Sanidad, Inocuidad y Calidad Agroalimentaria (SENASICA) *National Service of Health, Safety and Food Quality.* Mexican Government. Last access: December 2022 From: https://www.gob.mx/senasica/articulos/pequenos-productores-sosten-de-nuestra-agricultura

Schejtman, A. (1982) Economía campesina y agricultura empresarial. Tipología de productores del agro mexicano. CEPAL, Siglo XXI editores. México. Last access: december 2022, from: https://repositorio.cepal.org/handle/11362/25265

SIAP (2022). Information System of Agrifood and Fisheries (Sistema de Información Agroalimentaria y Pesquera SIAP). Ministry of Agriculture (Secretaría de Agricultura). Mexican Government. Last access: December 2022, From: https://www.gob.mx/siap

Toledo, V., Alarcón-Chaires, P., & Barón, L. (2002). *La modernización rural de México: un análisis socioecológico*. UNAM: México

UNCTAD (2015) *Commodities and Development Report 2015: Smallholder Farmers and Sustainable Commodity Development UNCTAD/SUC/2014/5,* United Nations Conference on Trade and Development, Geneva, Switzerland. Last access: December 2022, from: unctad.org/en/PublicationsLibrary/suc2014d5_en.pdf

Vargas Canales, J. M., Palacios Rangel, M. I., Camacho Vera, J. H., Aguilar Ávila, J., & Ocampo Ledesma, J. G. (2015). Factores de innovación en agricultura protegida en la región de Tulancingo, México. Revista mexicana de ciencias agrícolas, 6(4), 827-840. https://www.scielo.org.mx/scielo.php?script=sci_arttext&pid=S2007-09342015000400013

Yúnez, A., Cisneros, A. & Meza, P. (2013) Situando la agricultura familiar en México. Principales características y tipología. Serie Documentos de Trabajo N°149. Grupo de Trabajo: Desarrollo con Cohesión Territorial. Programa Cohesión Territorial para el Desarrollo. Rimisp, Santiago, Chile. Last access: december 2022, from: https://www.rimisp.org/documentos/situando-la-agricultura-familiar-en-mexico-principales-caracteristicas-y-tipologia/

Wiggins S, Kirsten J., & Llambi L. (2010) The future of small farms *World Development.* 38 1341–8. https://doi.org/10.1016/j.worlddev.2009.06.013

World Bank 2007 *World Development Report 2008: Agriculture for Development* (Washington, DC: The World Bank). Last access: December 2022, From: https://elibrary.worldbank.org/doi/abs/10.1596/978-0-8213-6807-7

Appendix 1: Mexican agricultural production and domestic supply of the 29 agricultural products analysed in this study in million tonnes, for the year 2019, compared with the total values reported by the FAO. Source of data: calculations by the authors using FAO (2022c) for agricultural production values (data of "crops primary" and "livestock primary"), and FAO (2022a) for domestic supply values. Note that FAO does not give values for amaranth and alfalfa. [23] The details on the values with decimal points are shown in the Supplementary Information.

Table A1. Agricultural Production

Name of agricultural product according to each data source		Production [Million tons]
**Crops and livestock products (FAO, 2022c)**	**INEGI (2019)**<br>(food group used in this study)	
Maize (corn)	white & yellow maize	27.23
Wheat	wheat	3.24
Rice	rice	0.25
Amaranth	amaranth	no data
Beans, dry	bean	0.88
Pumpkins, squash and gourds	squash	0.82
Onions and shallots, dry (excluding dehydrated)	onion	1.49
Chillies and peppers, green (Capsicum spp. and Pimenta spp.)	chilli pepper	2.72
Tomatoes	tomato	4.27
Avocados	avocado	2.30
Strawberries	strawberry	0.86
Lemons and limes	lemon	2.70
Mangoes, guavas and mangosteens	mango	2.40
Apples	apple	0.76
Oranges	orange	4.74
Bananas	banana	2.40
Grapes	grape	0.47
Sorghum	sorghum	4.35
Soya beans	soybean	0.23
Raw milk of cattle	milk	12.28
Meat of cattle with the bone, fresh or chilled	[12]cattle meat	2.03

Meat of pig with the bone, fresh or chilled	pork	1.60
Meat of chickens, fresh or chilled	chicken meat	3.48
Hen eggs in shell, fresh	eggs	2.95
sugar cane	sugar cane	59.33
Coffee, green	coffee	0.17
Cocoa beans	cocoa	0.03
TOTAL for the 29 agricultural products included in this study:		143.97
Total "crops primary" and "livestock primary" reported by the FAO (2022c)		166.97
Share of the 29 food products included in this study in Mexico’s total agricultural production		87%

Table A2. Domestic supply:

**Name of agricultural product according to each data source**		**[Million tons]**
**Food Balance Sheets (FAO, 2022a)**	**INEGI (2019) (food group used in this study)**	**Domestic supply** [Million tons]
Wheat and products	wheat	6.84
Rice and products	rice	1.41
Maize and products	white and yellow maize	47.13
Beans	bean	1.11
Tomatoes and products	tomato	2.56
Onions	onion	1.27
Vegetables, other	chilli pepper, squash	5.91
Lemons, Limes and products	lemon	1.73
Apples and products	apple	0.93
Oranges, Mandarines	orange	4.63
Bananas	banana	1.83
Grapes and products (excl wine)	grape	0.39
Fruits, other	avocado, strawberry, mango	6.11
Sugar cane	sugar cane	59.33

Coffee and products	coffee	0.08
Cocoa Beans and products	cocoa	0.09
Sorghum and products	sorghum	5.20
Soyabeans	soybean	4.78
Bovine Meat	[12]cattle meat	1.90
Pigmeat	pork	2.34
Poultry Meat	chicken meat	4.65
Eggs	eggs	3.00
Milk - Excluding Butter	milk	13.49
TOTAL amount in tonnes included in this study		176.73
Total domestic supply reported by the FAO (2022a)		210.44
Share of total included in this study		84%

[22] **Appendix 2**. Market and non-market production by each type of farm and each type of crop. Calculations by the authors using INEGI (2019a)

The following tables show the share (per farm and crop) of the production and the total production in tons that was sold ("market") and not sold ("no market") by the farms. The "no market" production includes the seeds that farms use for the following farming season, the food used for home consumption, and the feed used for livestock. [23] The details of the share and production for seeds, food and feed, as well as the values with decimal points, are shown in the Supplementary Information.

Table A3. Total production of no market and market production produced by each type of farm and each type of crop. Source of data: calculations by the authors using INEGI (2019a)

Tons	Small farms		Medium farms		Large farms	
	Not market	market	Not market	market	Not market	market
Avocado	135	743,790	569	823,376	1,687	445,549
Alfalfa	863,822	1,694,021	171,608	1,324,157	484,387	1,070,391
Amaranth	8	567	69	1,020	223	3,466
Rice	868	107,858	183	39,057	23	9,443
Cocoa	46	3,229	1,192	5,407	1,749	12,644
Coffee	576	102,597	4,052	147,556	12,465	213,991
Squash	1,108	393,283	399	124,937	287	109,682
Sugar Cane	45,596	14,855,458	42,287	24,000,000	65,156	14,337,786
Onion	50	826,122	9	129,075	114	43,953
Chilli pepper	449	800,517	1,019	604,318	574	47,899
Strawberry	-	21,574	4	14,085	14	8,085
Bean	44,175	465,147	40,011	226,473	17,751	34,507
Tomato	907	893,758	1,265	121,576	162	57,949
Lemon	279	912,147	131	307,623	573	130,303
Yellow maize	521,165	3,500,598	247,125	694,573	197,026	240,494
White maize	1,231,565	10,724,702	1,152,651	8,453,322	1,261,406	2,987,188
Mango	325	473,256	2,584	253,098	1,152	184,598
Apple	157	222,431	5	147,663	223	22,376
Orange	1,370	1,617,578	3,261	1,232,379	3,148	642,371
Banana	438	1,499,405	555	140,201	1,296	285,591
Sorghum	193,156	2,199,371	56,589	869,511	13,056	339,386
Soybean	674	271,210	303	33,767	0	4,458
Wheat	16,881	3,328,673	2,338	367,088	280	19,831
Grape	47	283,532	15	20,745	-	-

Table A4. Share of no market and market production produced by each type of farm and each type of crop

	Small farms		Medium farms		Large farms	
	No market	market	No market	market	No market	market
Avocado	0.02%	99.98%	0.07%	99.93%	0.38%	99.62%
Alfalfa	33.77%	66.23%	11.47%	88.53%	31.15%	68.85%
Amaranth	1.33%	98.67%	6.33%	93.67%	6.04%	93.96%
Rice	0.80%	99.20%	0.47%	99.53%	0.24%	99.76%
Cocoa	1.39%	98.61%	18.06%	81.94%	12.15%	87.85%

Coffee	0.56%	99.44%	2.67%	97.33%	5.50%	94.50%
Squash	0.28%	99.72%	0.32%	99.68%	0.26%	99.74%
Sugar Cane	0.31%	99.69%	0.18%	99.82%	0.45%	99.55%
Onion	0.01%	99.99%	0.01%	99.99%	0.26%	99.74%
Chilli pepper	0.06%	99.94%	0.17%	99.83%	1.18%	98.82%
Strawberry	0.00%	100.00%	0.03%	99.97%	0.17%	99.83%
Bean	8.67%	91.33%	15.01%	84.99%	33.97%	66.03%
Tomato	0.10%	99.90%	1.03%	98.97%	0.28%	99.72%
Lemon	0.03%	99.97%	0.04%	99.96%	0.44%	99.56%
Yellow maize	12.96%	87.04%	26.24%	73.76%	45.03%	54.97%
White maize	10.30%	89.70%	12.00%	88.00%	29.69%	70.31%
Mango	0.07%	99.93%	1.01%	98.99%	0.62%	99.38%
Apple	0.07%	99.93%	0.00%	100.00%	0.99%	99.01%
Orange	0.08%	99.92%	0.26%	99.74%	0.49%	99.51%
Banana	0.03%	99.97%	0.39%	99.61%	0.45%	99.55%
Sorghum	8.07%	91.93%	6.11%	93.89%	3.70%	96.30%
Soybean	0.25%	99.75%	0.89%	99.11%	0.00%	100.00%
Wheat	0.50%	99.50%	0.63%	99.37%	1.39%	98.61%
Grape	0.02%	99.98%	0.07%	99.93%	0.00%	0.00%

Appendix 3. Imports and exports of agricultural products estimated for this study. Calculations by the authors using INEGI (2019a) and FAO (2022b).

The FAO gives data of exports and imports (FAO, 2022b) and production values (FAO, 2022c) of all primary products. The values of production (FAO, 2022c) are slightly different from the values reported by the INEGI (2019a) (values of this paper) due to differences in methodology and type of agricultural products used in each data source. For this reason, for this study we calculated a "ratio value" of exports and imports using FAO data (table A4). This ratio was used to calculate the exports and imports for the 29 food groups of this study. Table A5 shows the results of this calculation.

The FAO does not distinguish between white and yellow maize. So, yellow and white maize production and trade values were taken from SAGARPA (2017). [23] The details on the values with decimal points are shown in the Supplementary Information.

Table A5. Estimation of exports and imports ratio value. Data source: Production (FAO, 2022c), imports and exports (FAO 2022b).

**Name of agricultural product according to each data source**		Production (FAO, 2022c)	Exports (FAO 2022b)	Imports (FAO 2022b)	Ratio of exports	Ratio of imports
**FAO (2022b,2022c)**	**INEGI (2019)** (food group used in this study)					
	White maize	24.56	1.499	1.066	0.06	0.04
Wheat	Wheat	3.24	0.74	4.80	0.23	1.48
Rice, paddy (rice milled eq)	Rice	0.16	0.01	0.97	0.06	5.95
	Amaranth	no data	no data	no data	-	-
beans, dry	Bean	0.88	0.04	0.12	0.05	0.14
sugar cane	Sugar cane	no data	no data	no data	-	-
Pumpkins, squash and gourds	Squash	0.68	0.54	0.00	0.79	-
Onions, dry	Onion	1.49	0.35	0.13	0.24	0.09
Chillies and peppers, green	Chili pepper	3.24	1.07	0.00	0.33	0.00
Tomatoes	Tomato	4.27	1.86	0.00	0.44	0.00
Avocados	Avocado	2.30	1.15	0.00	0.50	-
Strawberries	Strawberry	0.86	0.14	0.02	0.16	0.02
Lemons and limes	Lemon	2.70	0.77	0.00	0.28	0.00
Mangoes, mangosteens, guavas	Mango	2.40	0.41	0.00	0.17	0.00
Apples	Apple	0.76	0.00	0.25	0.00	0.33
Oranges	Orange	4.74	0.06	0.03	0.01	0.01
Bananas	Banana	2.40	0.57	0.00	0.24	0.00

Grapes	Grape	0.47	0.22	0.09	0.46	0.19
Cocoa, beans	Cocoa	0.03	0.00	0.05	0.00	1.64
Coffee, green	Coffee	0.17	0.10	0.04	0.59	0.24
	Yellow maize	3.55	0.035	12.95	0.01	3.65
Alfalfa meal and pellets	Alfalfa	0.0	0.0	0.0	0.0	0.0
Sorghum	Sorghum	4.35	0.00	0.74	0.00	0.17
Soybeans	Soybean	0.23	0.00	4.85	0.01	20.85
Milk, whole fresh cow	Milk	12.28	0.00	0.21	0.00	0.02
Meat, cattle	[12]Cattle meat	2.03	0.10	0.00	0.05	0.00
Meat, pig	Pork	1.60	0.00	0.70	0.00	0.44
Meat, chicken	Chicken meat	3.48	0.00	0.97	-	0.28
Eggs, hen, in shell	Eggs	2.95	0.00	0.05	-	0.02

Table A6. Production, exports, imports, and domestic supply values. Data source: Calculations by the authors.

**[Million tons]**	Production	Exports	Imports	Domestic Supply
White maize	25.81	1.58	1.12	25.36
Wheat	3.74	0.85	5.53	8.42
Rice	0.16	0.01	0.94	1.08
Amaranth	0.01	0.00	0.00	0.01
Bean	0.83	0.04	0.12	0.90
Sugar cane	53.25	0.00	0.00	53.25
Squash	0.64	0.50	0.00	0.13
Onion	1.00	0.24	0.09	0.85
Chilli pepper	2.00	0.66	0.00	1.34
Tomato	2.86	1.24	0.00	1.62
Avocado	2.02	1.01	0.00	1.01
Strawberry	0.23	0.04	0.00	0.20
Lemon	1.35	0.38	0.00	0.97
Mango	0.92	0.16	0.00	0.76
Apple	0.48	0.00	0.16	0.64
Orange	3.50	0.04	0.02	3.48

Banana	1.93	0.46	0.00	1.47
Grape	0.32	0.15	0.06	0.23
Cocoa	0.02	0.00	0.04	0.06
Coffee	0.48	0.29	0.12	0.31
Yellow maize grain	5.40	0.05	19.70	25.05
Alfalfa	5.61	0.00	0.00	5.61
Sorghum	3.67	0.00	0.63	4.30
Soybean	0.31	0.00	6.48	6.78
Milk	12.49	0.00	0.21	12.70
[12]Cattle meat	2.03	0.10	0.00	1.93
Pork	1.60	0.00	0.70	2.30
Chicken meat	3.48	0.00	0.97	4.45
Eggs	2.95	0.00	0.05	3.00
TOTAL	139.08	7.80	36.95	168.22

Appendix 4. Distribution of agricultural products production by type of farm in Mexico.

Values are given in million tons, the share of the production per type of farms indicated in parenthesis. Source: calculations by the authors. See section 3.1 for details. [23]

Table A7. Distribution of agricultural products production by type of farm in Mexico.

Mt (%)	Small scale	Medium scale	Large scale	Protected agriculture	Total
White maize	4.0 (17%)	9.0 (37%)	11.2 (46%)	0.0 (0%)	24.2 (100%)
Other cereals	0.0 (1%)	0.3 (11%)	2.7 (88%)	0.0 (0%)	3.0 (100%)
Beans	0.0 (6%)	0.3 (32%)	0.5 (62%)	0.0 (0%)	0.8 (100%)
Vegetables	0.1 (3%)	0.6 (15%)	1.8 (46%)	1.4 (36%)	3.8 (100%)
Fruits	1.4 (16%)	2.3 (28%)	4.6 (53%)	0.2 (3%)	8.5 (100%)
Animal feed	2.3 (16%)	3.4 (23%)	9.2 (61%)	0.0 (0%)	14.9 (100%)
Animal products	3.2 (14%)	3.9 (18%)	15.3 (68%)	N.A.	22.4 (100%)
Stimulants	0.1 (48%)	0.1 (31%)	0.0 (20%)	0.0 (1%)	0.2 (100%)
Sugar cane	14.3 (27%)	24.0 (45%)	14.9 (28%)	0.0 (0%)	53.3 (100%)
TOTAL	25.6 (19%)	43.9 (33%)	60.1 (46%)	1.6 (2%)	131.3 (100%)

General response to reviewers:

We have received useful comments from two anonymous reviewers. Based on those, we have made a major revision to the manuscript mainly on clarifying the source of the raw data used in our analysis, and the methodology. Also, we have made English editing.

Below, we add in black the questions of the review process and the responses of the two anonymous reviewers. In blue, we respond to each of the comments of both reviewers. At the end, we add the comments of reviewer 2 which were written within the pdf manuscript. We respond to those comments in blue.

In the new version of the manuscript, called "manuscript track changes", we highlight in yellow the changes based on the reviewers' comments. To identify the changes with each comment of the reviewers, we number between brackets each comment and add that numbering in the manuscript highlighted in blue. We also attach a "clean manuscript" with no tracking changes.

<u>Reviewer's Responses to Questions</u>

1. Is the manuscript technically sound, and do the data support the conclusions?

The manuscript must describe a technically sound piece of scientific research with data that supports the conclusions. Experiments must have been conducted rigorously, with appropriate controls, replication, and sample sizes. The conclusions must be drawn appropriately based on the data presented.

Reviewer #1: Yes Thanks

Reviewer #2: Partly. Hope that the new version of the manuscript is more technically sound. If you think otherwise, we would like to know in what specific aspects you think the manuscript is failing to improve it.

2. Has the statistical analysis been performed appropriately and rigorously?

Reviewer #1: I Don't Know

Reviewer #2: No

[1] Response to both reviewers: We believe that the negative response of both reviewers to this question was because the first version of the manuscript did not explain in detail calculations performed to estimate the total agricultural production per type of farm. We also failed to present in sufficient detail the source of the data (the microdata of the national survey of agriculture). The national survey of agriculture was applied to a statistically representative sample of farms. The sample of farms is designed to be representative at a national scale for the 29 agricultural products used

in this study, as well as for the type of farms used in the study (small, medium, and large scale farms). We validated our results by comparing the total values estimated in our study with the total values reported by the INEGI (2019). We did not perform a statistical analysis, but the fact that our values are based on the validated data of the national survey implies that our results are robust and statistically appropriate. In the new version of the manuscript, we describe in more detail the type of data of the national survey and the methodological steps to estimate our results. If you believe that still this is not clear, of the calculations are not appropriate, we would be grateful if you can describe in detail what is missing.

3. Have the authors made all data underlying the findings in their manuscript fully available?

The PLOS Data policy requires authors to make all data underlying the findings described in their manuscript fully available without restriction, with rare exception (please refer to the Data Availability Statement in the manuscript PDF file). The data should be provided as part of the manuscript or its supporting information, or deposited to a public repository. For example, in addition to summary statistics, the data points behind means, medians and variance measures should be available. If there are restrictions on publicly sharing data—e.g. participant privacy or use of data from a third party—those must be specified.

Reviewer #1: Yes

Reviewer #2: No [2] In the new version of the manuscript, we have mentioned that the raw data come from a national survey, which are not public due to confidentiality of personal data, but are available upon request to INEGI. The public data are the summarized statistics available in www.inegi.org.mx (specifically at: https://www.inegi.org.mx/programas/ena/2019/#tabulados).. However, by sharing the details on the calculations in the new version, we believe that the source of information for our calculations is clear. Also, we have included the Supplementary Information with the detail values (including all decimal points) of all tables and figures of the paper (see point comments point [23]). If you believe that something is missing, we would appreciate it if you can comment on what is missing.

4. Is the manuscript presented in an intelligible fashion and written in standard English?

PLOS ONE does not copyedit accepted manuscripts, so the language in submitted articles must be clear, correct, and unambiguous. Any typographical or grammatical errors should be corrected at revision, so please note any specific errors here.

Reviewer #1: Yes

Reviewer #2: Yes

5. Review Comments to the Author

Please use the space provided to explain your answers to the questions above. You may also include additional comments for the author, including concerns about dual publication, research ethics, or publication ethics. (Please upload your review as an attachment if it exceeds 20,000 characters)

Reviewer #1: In this manuscript (MS) the respected authors have written about the contributions of small farms to the Mexican food industry. The MS contains several citations relevant to the topic discussed. The authors cite documents supporting and opposing their theory.

Major issues: none

Minor issues:

There are some minor typoes and missing punctuation marks at the end of a sentences.

In the PDF format Figure 1 is pixelated. I'm not sure if this is caused by the editorial system (e.g. downscaling the resolution) or the original image was less than 300 DPI in quality. We think the problem is that it is "inserted" into the word document. And then the resolution downscales when the journal's system converts it to pdf. We added the figure as a separate file.

The english of the Materials and methods section could be improved. [3] We have improved this section

Please correct the name of Schetjman to Schejtman. [4] Done, thanks.

Line 90: FAO, 2014 is not listed in references. [5] Thanks for point this out. The correct reference for this line is "FAO, 2018". We have corrected it.

In line 131 the correct citation is: (Bustamante et al., 2017) [6] Corrected.

In line 137: (Sánchez, 2003) [7] Corrected.

Line 186: correctly: de Ita [8] Corrected.

In line 220 I would use beef cows or cows for meat production instead of "fattening cows". In lines 222 and 223, and later on instead of "fattening" I'd rather use the expression "for meat production". [9] Good idea. Changed.

In line 246 instead of "recycled seeds" I'd suggest to use "farm saved seeds". [10] Good idea. Changed.

Also in line 247 "improved" seeds should be rephrased to correctly specify/clarify the meaning. ". [11] We have clarified the meaning of "improved seeds" in parenthesis.

Line 253 use "fodder crops" instead of "feed crops". Line 271 farms in plural at the end of the sentence. Line 277 use "fodder crops" instead of "animal feed". Line 286 use "cattle" instead of "bovine".[12] Corrected.

I would suggest explaining why protected growing is more efficient than other production methods. For example in protected cultivation plants could be grown year round, and because more of the environmental factors can be controlled. Also it would be worth mentioning that the cultivation there is more intensive, and growers use high yielding cultivars/seeds. [13] Good idea. We have added your suggestion, and included a reference to support it.

In section 3.2 the authors write about "chemical fertilizers". I was wondering if this could be rephrased in the MS as "inorganic fertilizers" as in my opinion it describes them more accurately. [14] Good idea. We have changed it throughout the text.

Also in this section "organic fertilizers" need clarification. Does this phrase mean that these fertilizers are certified Organic by some authorities or this just means that these fertilizers are animal byproducts (like manure) and compost? [15] We have indicated what type of inorganic and organic fertilizers are reported by the farmers based on the questionnaire of the national survey.

Line 343: FAOSTAT, 2019 is not listed in references. [16] Corrected

Line 365 add a space after the comma: "cropland, 16 pigs ...". [17] Corrected

Line 403, please omit "robust". [18] Deleted

Line 525 correctly: de Ita, ... [19] Corrected

Lines 421 and 540: What is the correct name of the Author, Leff or Left? Please change it both places to match. [20] It is Leff. Corrected. Thanks

Line 549: Lowder, S. K., Sánchez, M. V., & Bertini, R. (2021) is not cited in the text. [21] Corrected

Reviewer #2: This is a very interesting subject matter especially for developing countries agricultural economies. My first worry with this research is the fact that it did not take into account stable crop production. This means that important production for home consumption and gifts has been neglected somehow and therefore the results of this analysis might not be accurate. This may explain the reason why their results are much lower than the national reported estimates. I will suggest they look for a way to include this data either through a case study or so and redo the analysis.

[22] Thanks for this comment. This made us realize that our source of data was not well described. In the new version, we describe the production data in more detail. All production for self consumption (seeds, food and feed for the farm's livestock) is

included in the production data. We realized that it might be of interest to some readers to indicate the share of this production. Therefore, we have included Appendix 4 and the details in the Supplementary Information. This appendix includes the share of the production, for each farm and each crop, (1) which is sold, and (2) which is used by the farm as seeds, food or feed. We also included a paragraph in the results section briefly describing this.

Elsewhere, some of the percentages did not sum up to 100 meaning there are some problems with the calculations

[23] Thanks for pointing this out. We realized that we have rounded up the values to not have decimal points in the percentages, but we missed some of the decimals due to the rounding. We have corrected this, the sum of all lines is corrected in Table 4. In addition, we add the Supplementary Information with the values of all the tables and figures of the paper with all the decimal numbers.

Comments of reviewer two written in the manuscript:

In the abstract: This is strange that small farms do not contribute to staple food production whereas I this this s even the motive of most small farms. I wish to find out if it was that the data base did not capture this aspect because of its complexity or it is that small farms don't produce staple crops. And if it is that small farms produce staple crops, then the percentage of their contribution will obviously be higher. The authors need to reinvestigate and clarify this aspect. [24] We realized the description of our results was not clear. In the abstract, we have specified that, when analyzing the total production of small farms, it is mainly cash crops, and to a lesser extend, staple crops (maize and beans). You are right, small farms produce staple crops. But, as our findings show and we believe that it is an important result, that in total, small farms produce in its majority these cash crops.

In Table 3: I think this data is not sufficient to explain the production and contribution of small scale farms to the overall production in Mexico. This is because production for self consumption, gifts and informal trade has not been accounted for in the over all data.
This has been solved and discussed in the new version. See comment [22]

In Table 4: The total in many lines is not 100%, is less or more (highlighted in yellow)
This has been corrected. See comment [23]
