# Supplementary material for: How much of the Mexican agricultural supply is produced by small farms, and how?
Source: PLoS One. 2023 Oct 5;18(10):e0292528. doi: 10.1371/journal.pone.0292528 (PMC10553241; doi:10.1371/journal.pone.0292528)
Supplement: Attachment — Submitted filename: response to reviewers.docx [file pone.0292528.s007.docx]

**General response to reviewers:**

We have received useful comments from two anonymous reviewers. Based on those, we have made a major revision to the manuscript mainly on clarifying the source of the raw data used in our analysis, and the methodology. Also, we have made English editing.

Below, we add in black the questions of the review process and the responses of the two anonymous reviewers. In blue, we respond to each of the comments of both reviewers. At the end, we add the comments of reviewer 2 which were written within the pdf manuscript. We respond to those comments in blue.

In the new version of the manuscript, called “manuscript track changes”, we highlight in yellow the changes based on the reviewers’ comments. To identify the changes with each comment of the reviewers, we number between brackets each comment and add that numbering in the manuscript highlighted in blue. We also attach a “clean manuscript” with no tracking changes.

***Reviewer's Responses to Questions***

1. Is the manuscript technically sound, and do the data support the conclusions?

The manuscript must describe a technically sound piece of scientific research with data that supports the conclusions. Experiments must have been conducted rigorously, with appropriate controls, replication, and sample sizes. The conclusions must be drawn appropriately based on the data presented.

Reviewer #1: Yes Thanks

Reviewer #2: Partly. Hope that the new version of the manuscript is more technically sound. If you think otherwise, we would like to know in what specific aspects you think the manuscript is failing to improve it.

2. Has the statistical analysis been performed appropriately and rigorously?

Reviewer #1: I Don't Know

Reviewer #2: No

[1] Response to both reviewers: We believe that the negative response of both reviewers to this question was because the first version of the manuscript did not explain in detail calculations performed to estimate the total agricultural production per type of farm. We also failed to present in sufficient detail the source of the data (the microdata of the national survey of agriculture). The national survey of agriculture was applied to a statistically representative sample of farms. The sample of farms is designed to be representative at a national scale for the 29 agricultural products used in this study, as well as for the type of farms used in the study (small, medium, and large scale farms). We validated our results by comparing the total values estimated in our study with the total values reported by the INEGI (2019). We did not perform a statistical analysis, but the fact that our values are based on the validated data of the national survey implies that our results are robust and statistically appropriate. In the new version of the manuscript, we describe in more detail the type of data of the national survey and the methodological steps to estimate our results. If you believe that still this is not clear, of the calculations are not appropriate, we would be grateful if you can describe in detail what is missing.

3. Have the authors made all data underlying the findings in their manuscript fully available?

The PLOS Data policy requires authors to make all data underlying the findings described in their manuscript fully available without restriction, with rare exception (please refer to the Data Availability Statement in the manuscript PDF file). The data should be provided as part of the manuscript or its supporting information, or deposited to a public repository. For example, in addition to summary statistics, the data points behind means, medians and variance measures should be available. If there are restrictions on publicly sharing data—e.g. participant privacy or use of data from a third party—those must be specified.

Reviewer #1: Yes

Reviewer #2: No [2] In the new version of the manuscript, we have mentioned that the raw data come from a national survey, which are not public due to confidentiality of personal data, but are available upon request to INEGI. The public data are the summarized statistics available in [www.inegi.org.mx](http://www.inegi.org.mx) (specifically at: <https://www.inegi.org.mx/programas/ena/2019/#tabulados>).. However, by sharing the details on the calculations in the new version, we believe that the source of information for our calculations is clear. Also, we have included the Supplementary Information with the detail values (including all decimal points) of all tables and figures of the paper (see point comments point [23]). If you believe that something is missing, we would appreciate it if you can comment on what is missing.

4. Is the manuscript presented in an intelligible fashion and written in standard English?

PLOS ONE does not copyedit accepted manuscripts, so the language in submitted articles must be clear, correct, and unambiguous. Any typographical or grammatical errors should be corrected at revision, so please note any specific errors here.

Reviewer #1: Yes

Reviewer #2: Yes

5. Review Comments to the Author

Please use the space provided to explain your answers to the questions above. You may also include additional comments for the author, including concerns about dual publication, research ethics, or publication ethics. (Please upload your review as an attachment if it exceeds 20,000 characters)

**Reviewer #1:** In this manuscript (MS) the respected authors have written about the contributions of small farms to the Mexican food industry. The MS contains several citations relevant to the topic discussed. The authors cite documents supporting and opposing their theory.

Major issues: none

Minor issues:

There are some minor typoes and missing punctuation marks at the end of a sentences.

In the PDF format Figure 1 is pixelated. I'm not sure if this is caused by the editorial system (e.g. downscaling the resolution) or the original image was less than 300 DPI in quality. We think the problem is that it is “inserted” into the word document. And then the resolution downscales when the journal’s system converts it to pdf. We added the figure as a separate file.

The english of the Materials and methods section could be improved. [3] We have improved this section

Please correct the name of Schetjman to Schejtman. [4] Done, thanks.

Line 90: FAO, 2014 is not listed in references. [5] Thanks for point this out. The correct reference for this line is “FAO, 2018”. We have corrected it.

In line 131 the correct citation is: (Bustamante et al., 2017) [6] Corrected.

In line 137: (Sánchez, 2003) [7] Corrected.

Line 186: correctly: de Ita [8] Corrected.

In line 220 I would use beef cows or cows for meat production instead of "fattening cows". In lines 222 and 223, and later on instead of "fattening" I'd rather use the expression "for meat production". [9] Good idea. Changed.

In line 246 instead of "recycled seeds" I'd suggest to use "farm saved seeds". [10] Good idea. Changed.

Also in line 247 "improved" seeds should be rephrased to correctly specify/clarify the meaning. ". [11] We have clarified the meaning of “improved seeds” in parenthesis.

Line 253 use "fodder crops" instead of "feed crops". Line 271 farms in plural at the end of the sentence. Line 277 use "fodder crops" instead of "animal feed". Line 286 use "cattle" instead of "bovine".[12] Corrected.

I would suggest explaining why protected growing is more efficient than other production methods. For example in protected cultivation plants could be grown year round, and because more of the environmental factors can be controlled. Also it would be worth mentioning that the cultivation there is more intensive, and growers use high yielding cultivars/seeds. [13] Good idea. We have added your suggestion, and included a reference to support it.

In section 3.2 the authors write about "chemical fertilizers". I was wondering if this could be rephrased in the MS as "inorganic fertilizers" as in my opinion it describes them more accurately. [14] Good idea. We have changed it throughout the text.

Also in this section "organic fertilizers" need clarification. Does this phrase mean that these fertilizers are certified Organic by some authorities or this just means that these fertilizers are animal byproducts (like manure) and compost? [15] We have indicated what type of inorganic and organic fertilizers are reported by the farmers based on the questionnaire of the national survey.

Line 343: FAOSTAT, 2019 is not listed in references. [16] Corrected

Line 365 add a space after the comma: "cropland, 16 pigs ...". [17] Corrected

Line 403, please omit "robust". [18] Deleted

Line 525 correctly: de Ita, ... [19] Corrected

Lines 421 and 540: What is the correct name of the Author, Leff or Left? Please change it both places to match. [20] It is Leff. Corrected. Thanks

Line 549: Lowder, S. K., Sánchez, M. V., & Bertini, R. (2021) is not cited in the text. [21] Corrected

**Reviewer #2:** This is a very interesting subject matter especially for developing countries agricultural economies. My first worry with this research is the fact that it did not take into account stable crop production. This means that important production for home consumption and gifts has been neglected somehow and therefore the results of this analysis might not be accurate. This may explain the reason why their results are much lower than the national reported estimates. I will suggest they look for a way to include this data either through a case study or so and redo the analysis.

[22] Thanks for this comment. This made us realize that our source of data was not well described. In the new version, we describe the production data in more detail. All production for self consumption (seeds, food and feed for the farm’s livestock) is included in the production data. We realized that it might be of interest to some readers to indicate the share of this production. Therefore, we have included Appendix 4 and the details in the Supplementary Information. This appendix includes the share of the production, for each farm and each crop, (1) which is sold, and (2) which is used by the farm as seeds, food or feed. We also included a paragraph in the results section briefly describing this.

Elsewhere, some of the percentages did not sum up to 100 meaning there are some problems with the calculations

[23] Thanks for pointing this out. We realized that we have rounded up the values to not have decimal points in the percentages, but we missed some of the decimals due to the rounding. We have corrected this, the sum of all lines is corrected in Table 4. In addition, we add the Supplementary Information with the values of all the tables and figures of the paper with all the decimal numbers.

**Comments of reviewer two written in the manuscript:**

**In the abstract:** This is strange that small farms do not contribute to staple food production whereas I this this s even the motive of most small farms. I wish to find out if it was that the data base did not capture this aspect because of its complexity or it is that small farms don't produce staple crops. And if it is that small farms produce staple crops, then the percentage of their contribution will obviously be higher. The authors need to reinvestigate and clarify this aspect. [24] We realized the description of our results was not clear. In the abstract, we have specified that, when analyzing the total production of small farms, it is mainly cash crops, and to a lesser extend, staple crops (maize and beans). You are right, small farms produce staple crops. But, as our findings show and we believe that it is an important result, that in total, small farms produce in its majority these cash crops.

**In Table 3:** I think this data is not sufficient to explain the production and contribution of small scale farms to the overall production in Mexico. This is because production for self consumption, gifts and informal trade has not been accounted for in the over all data.

This has been solved and discussed in the new version. See comment [22]

**In Table 4:** The total in many lines is not 100%, is less or more (highlighted in yellow)

This has been corrected. See comment [23]
