# Supplementary material for: How much of the Mexican agricultural supply is produced by small farms, and how?
Source: PLoS One. 2023 Oct 5;18(10):e0292528. doi: 10.1371/journal.pone.0292528 (PMC10553241; doi:10.1371/journal.pone.0292528)
Supplement: Attachment — Submitted filename: response reviewers.docx [file pone.0292528.s009.docx]

**General response:**

We have received useful comments from one anonymous reviewer. Based on those comments, we have improved the manuscript mainly by detailing the sample of the data source of this study: The National Agricultural Survey 2019 of Mexico.

Below, we add in black the questions of the review process and the responses of the two anonymous reviewers. In blue, we respond to each of the comments of both reviewers. At the end, we add the comments of reviewer 2 which were written within the pdf manuscript. We respond to those comments in blue.

In the new version of the manuscript, called “manuscript track changes”, we highlight in yellow the changes based on the reviewers’ comments. To identify the changes with each comment of the reviewers, we number between brackets each comment and add that numbering in the manuscript highlighted in blue. We also attach a “clean manuscript” with no tracking changes.

***Responses to Reviewers’ Questions and Comments***

1) There is a need for some text between figure 1 and Table 1

[1] We have moved Table 1 to the following page.

2) This paragraph is too short

[2] We have moved these sentences to the previous paragraph.

3) Provide some information on the methodology that the national institute of statistics used in collecting this data, the limitations of the approach and how this has been mitigated to increase reliability and validity

[3] We have expanded the text to better describe how INEGI gathered the data, and to present more specific data on the sample and universe sizes. Also, we noted that detailed information on this is specified in the methodological report of INEGI 2019b. The online link is in the references.

4) This table is confusing (Table A1). We have FAO; 2022 and INEGI but only one value? what does this mean? Yet the authors mention name of agricultural product according to each data source???

[4] Thank you for this observation. We realize this was confusing. The production data, and the name of the food category, of this table is from the FAO.. The “old” second column (which made the table confusing) indicated which of the food product of this paper is linked each of the FAO food product. We have removed the column with the names of the food products used in this paper, which are the ones used in inegi (2019), and better added them in italics and after a semicolon next to the name of the FAO food product.
